# Calpain cleavage of Junctophilin-2 generates a spectrum of calcium-dependent cleavage products and DNA-rich NT_1_-fragment domains in cardiomyocytes

**DOI:** 10.1038/s41598-022-14320-9

**Published:** 2022-06-20

**Authors:** Gunnar Weninger, Tatiana Pochechueva, Dana El Chami, Xiaojing Luo, Tobias Kohl, Sören Brandenburg, Henning Urlaub, Kaomei Guan, Christof Lenz, Stephan E. Lehnart

**Affiliations:** 1 Cellular Biophysics and Translational Cardiology Section, Heart Research Center Göttingen, University Medical Center Göttingen, Robert-Koch-Str. 42a, 37075 Göttingen, Germany; 2 Department of Cardiology and Pneumology, University Medical Center Göttingen, 37075 Göttingen, Germany; 3 Collaborative Research Center SFB1190 “Compartmental Gates and Contact Sites in Cells”, University of Göttingen, 37073 Göttingen, Germany; 4 Cluster of Excellence “Multiscale Bioimaging: from Molecular Machines to Networks of Excitable Cells” (MBExC2067), University of Göttingen, 37073 Göttingen, Germany; 5 DZHK (German Centre for Cardiovascular Research), partner site, 37075 Göttingen, Germany; 6 Institute of Pharmacology and Toxicology, Technische Universität Dresden, 01307 Dresden, Germany; 7 Proteomanalyse, Department of Clinical Chemistry, University Medical Center Göttingen, Robert-Koch-Str. 40, 37075 Göttingen, Germany; 8 Bioanalytical Mass Spectrometry, Max Planck Institute for Multidisciplinary Sciences, 37077 Göttingen, Germany; 9 Department of Physiology and Cellular Biophysics, Center for Molecular Cardiology, Columbia University Vagelos College of Physicians and Surgeons, New York, NY 10032 USA

**Keywords:** Biochemistry, Cell biology, Molecular biology, Stem cells, Cardiology, Molecular medicine

## Abstract

Calpains are calcium-activated neutral proteases involved in the regulation of key signaling pathways. Junctophilin-2 (JP2) is a Calpain-specific proteolytic target and essential structural protein inside Ca^2+^ release units required for excitation-contraction coupling in cardiomyocytes. While downregulation of JP2 by Calpain cleavage in heart failure has been reported, the precise molecular identity of the Calpain cleavage sites and the (patho-)physiological roles of the JP2 proteolytic products remain controversial. We systematically analyzed the JP2 cleavage fragments as function of Calpain-1 versus Calpain-2 proteolytic activities, revealing that both Calpain isoforms preferentially cleave mouse JP2 at R565, but subsequently at three additional secondary Calpain cleavage sites. Moreover, we identified the Calpain-specific primary cleavage products for the first time in human iPSC-derived cardiomyocytes. Knockout of RyR2 in hiPSC-cardiomyocytes destabilized JP2 resulting in an increase of the Calpain-specific cleavage fragments. The primary N-terminal cleavage product NT_1_ accumulated in the nucleus of mouse and human cardiomyocytes in a Ca^2+^-dependent manner, closely associated with euchromatic chromosomal regions, where NT_1_ is proposed to function as a cardio-protective transcriptional regulator in heart failure. Taken together, our data suggest that stabilizing NT_1_ by preventing secondary cleavage events by Calpain and other proteases could be an important therapeutic target for future studies.

## Introduction

Increased intracellular calcium (Ca^2+^) concentrations activate the neutral protease Calpain. In cardiomyocytes major isoforms exist, Calpain-1 and Calpain-2, which are ubiquitously expressed in other mammalian cell types^1^. Both isoforms form a complex with the regulatory Calpain subunit CAPNS1 (Calpain small subunit 1). Whereas Calpain-1 activation occurs at micromolar Ca^2+^ concentrations, higher millimolar concentrations are required to activate Calpain-2 under in vitro conditions^2,3^.

Both Calpain isoforms exhibit a high substrate specificity under varying conditions^4^. Following activation, Calpain translocates to distinct subcellular substrate locations to process its proteolytic target proteins, for example during cytoskeletal reorganization, plasma membrane repair or turnover of muscle sarcomeric proteins^5,6^. Major neurological and cardiac disorders have been associated with Calpain dysregulation and altered intracellular Ca^2+^ homeostasis including neuronal cell death due to ischemic brain injury^3^, cardiac cell death following myocardial infarction^7^, multiple sclerosis^8^, and Alzheimer's disease^9^.

In contrast to the protein erasing proteasomes and lysosomes as the major degradative proteolytic systems, Calpain cleaves its substrates through limited proteolysis steps. Hence, Calpain dependent substrate processing can generate cleavage products with additional biological functions. Interestingly, an N-terminal Calpain cleavage product of Junctophilin-2 (JP2), the major cardiac isoform, with unique cardio-protective functions has recently been discovered^10^.

**Figure 1:**
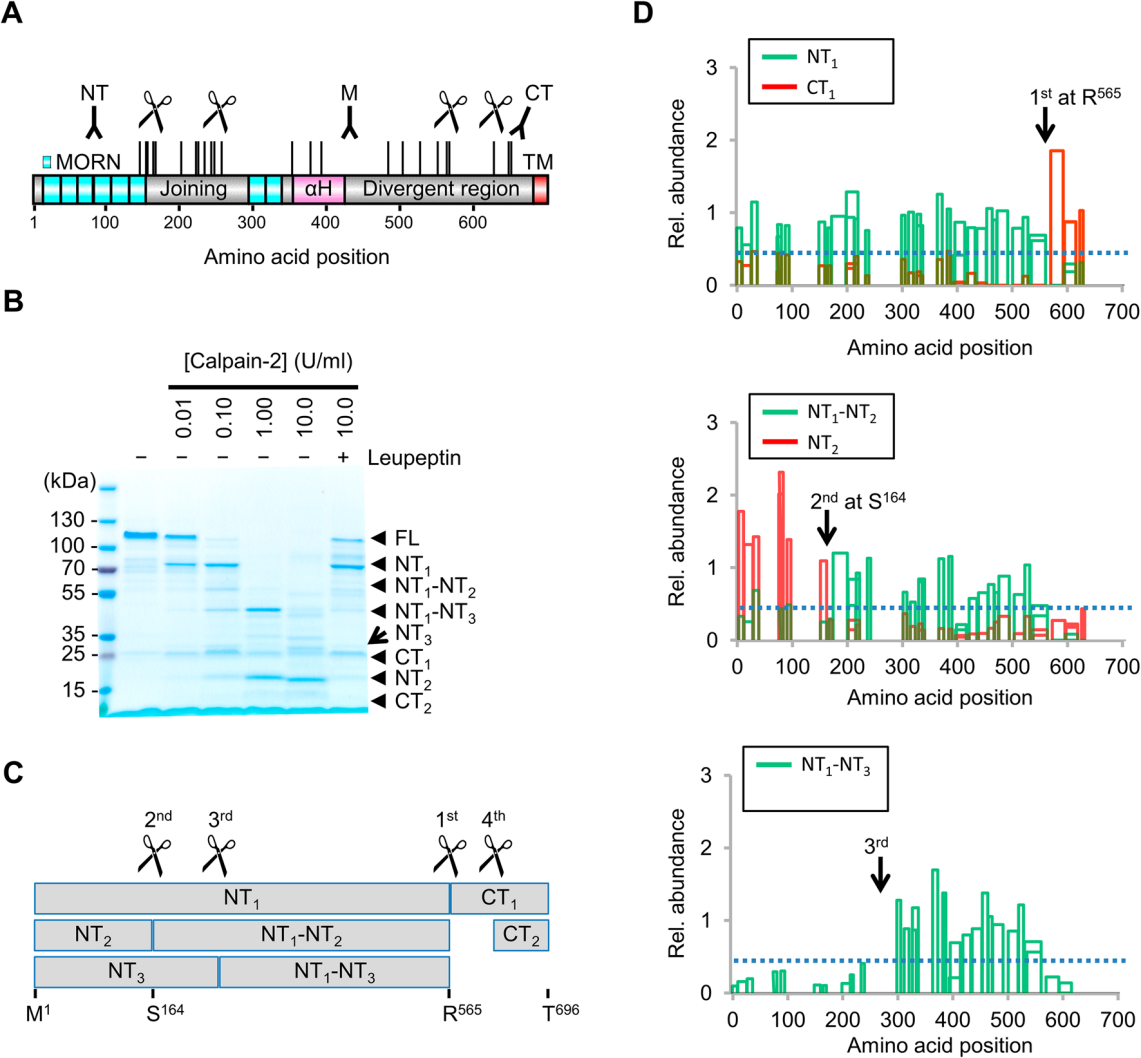
Identification of Calpain-specific cleavage fragments of JP2. (**A**) Mouse FL JP2 domain representation (Uniprot: Q9ET78) summarizing the in silico prediction of Calpain cleavage sites by the DeepCalpain tool. Predicted cleavage sites are indicated by vertical black lines above the JP2 topology domain model. Experimentally confirmed cleavage events observed at 0.1 U/ml Calpain-2 are indicated by scissors. The approximate epitope binding positions of three different JP2 antibodies are indicated by Y-shaped symbols: NT, N-terminal region; M, middle region; CT, C-terminal region. (**B**) The Calpain-2 activity specific cleavage patterns of the mouse FL JP2 substrate. Purified recombinant JP2 was incubated at increasing Calpain-2 concentrations for 30 min at 30 °C, followed by SDS-PAGE and Coomassie staining. (**C**) Box plot summarizing the JP2 cleavage events (scissors), fragments, and cleavage sites experimentally observed for the 0.1 U/ml Calpain-2 concentration. (**D**) LC-MS/MS analysis of the spectrum of mouse JP2 fragments generated by Calpain-2 digestion. Tryptic peptide counts are represented as relative abundance versus the JP2 amino acid position. Arrows indicate the positions of the first, second, and third cleavage events.

Full-length (FL) JP2 functions as a membrane tether, residing as a single-pass tail-anchored protein in the sarcoplasmic reticulum (SR) membrane in cardiomyocytes^11–13^. Bioinformatic analysis predicts eight highly conserved N-terminal MORN (membrane occupation and recognition nexus) motifs^11,14^, which provide the capacity for JP2 binding to the cytosolic membrane leaflet (Fig. 1A). Furthermore, the MORN and TM domains are connected by the α-helical and the divergent region (Fig. 1A), together bridging the discontinuous dyadic membrane contact subspace (≈15 nm) between the junctional SR and the Transverse (T-)tubule membrane invaginations in cardiomyocytes^15^ and between the SR and plasma membrane in smooth muscle cells^14^.

The molecular and spatial integrity of junctional membrane complexes is a key prerequisite for the vitally important functional coupling between voltage-gated L-type Ca^2+^ channels (LTCC) in T-tubules and ryanodine receptor 2 (RyR2) Ca^2+^ release channels during each heartbeat, the physiological process described as Ca^2+^ induced Ca^2+^ release (CICR)^16^. However, chronically activated Calpain can cleave JP2, disrupting the critical dyadic nanodomain spacing, which results in a loss of local CICR function in systole and increased diastolic SR Ca^2+^ leak^17^. Interestingly, in patient hearts with hypertrophic cardiomyopathy a decrease in FL JP2 occurs in the ventricular myocardium^18^. Accordingly, following transaortic banding and cardiac pressure overload in rodents the progression of heart failure correlates with decreased ventricular FL JP2 protein levels^13,19–21^.

While Calpain-specific JP2 cleavage fragments have been identified previously, the precise molecular spectrum of the cleavage sites and the identities of the proteolytic products remain controversial. Wu et al. introduced L201 as the first Calpain-specific JP2 cleavage site in a mouse model of heart failure associated with T-tubule reorganization and contractile loss-of-function^22^. Next, Song and colleagues identified a different Calpain cleavage site at R565, generating a stable N-terminal JP2 cleavage product, subsequently translocating into the cardiomyocyte nucleus, where it functions as a cardio-protective transcriptional repressor of reactive gene networks, which drive heart failure progression^10,23^. In contrast, Lahiri et al. identified the primary Calpain cleavage site at G482, generating a C-terminal JP2 cleavage fragment translocating into the cardiomyocyte nucleus in the failing mouse heart^24^.

While cardio-protective Calpain-specific cleavage products raise expectations for therapeutic targeting in heart disease^10,24^, only state-of-the-art unbiased proteomic approaches can solve the current controversy about the primary JP2 cleavage site^10,22–24^. Here, we investigate the spectrum and identities of the proteolytic Calpain-1 and Calpain-2 cleavage cascade, identifying the primary and secondary JP2 substrate fragmentation products in vitro and in living cardiomyocytes. Furthermore, we report the first systematic molecular JP2 fragmentation mapping for Calpain-1 and Calpain-2 in mouse and human cardiomyocytes.

## Results

### Characterization of JP2 fragmentation products following Calpain-1 or Calpain-2 proteolysis

Previously, immunoblotting against different JP2 epitopes or fusion tagging have been applied to identify Calpain-specific JP2 proteolytic fragments in cell lysates^17,22–24^. However, because of alternative proteolytic pathways, species-limited and affinity-biased antibody choices and the anomalous migration of JP2 in SDS-PAGE gels, the spectrum of JP2 cleavage fragments and sites remains incompletely characterized and hence uncertain. An in silico analysis with the computational tool DeepCalpain^25^ indeed predicts multiple additional Calpain cleavage sites in the joining and divergent regions of JP2 (Fig. 1A), and thus suggests previously unknown cleavage fragments. To investigate Calpain dependent JP2 proteolysis in a systematic and directly controlled substrate-reaction manner, we expressed recombinant mouse FL JP2 in *E. coli* followed by protein purification. Following molecular confirmation of FL JP2 by immunoblotting and LC-MS/MS (data not shown), the FL JP2 substrate was exposed to purified Calpain-1 (C6108, Sigma-Aldrich) or Calpain-2 (#208715, Calbiochem) throughout four orders of neutral protease concentrations (0.01 through 10 U/ml) to identify the spectrum of cleavage-specific products under constant reaction conditions.

In order to determine the apparent molecular mass of the Calpain-2 specific JP2 fragments, we used Coomassie Blue R250 as sensitive total protein stain to capture the complete cleavage pattern throughout increasing protease concentrations (Fig. 1B). At the lowest Calpain-2 concentration (0.01 U/ml), we observed a single major cleavage event resulting in two JP2 cleavage products as expected. The corresponding N- and C-terminal fragments denominated NT_1_ and CT_1_ migrate at apparent molecular weights (MWs) of ~75 kDa and ~25 kDa, respectively (Fig. 1B). At the next 10-fold higher Calpain-2 concentration (0.1 U/ml), three additional cleavage events are evident: two N-terminal cleavage reactions produce two additional fragment pairs (i) NT_2_ and NT_1_-NT_2_ with MWs of ~20 kDa and ~55 kDa, respectively; and (ii) NT_3_ and NT_1_-NT_3_ with MWs of ~30 kDa and ~45 kDa, respectively; C-terminally (iii) CT_1_ is cleaved and detected as a ~10 kDa fragment CT_2_. Notably, the computationally predicted CT_1_-CT_2_ and NT_3_-NT_2_ fragments were not detected (Fig. 1B,C). Moreover, the 100-fold higher Calpain-2 concentration (1 U/ml) generates the fragments NT_2_ and NT_1_-NT_3_, while CT_2_ remains stable and was thus not further cleaved. The highest Calpain-2 concentration (10 U/ml) results in an apparent shift of the remaining most abundant secondary cleavage product NT_2_ to a slightly smaller molecular mass, indicating a tertiary step of proteolytic processing (Fig. 1B, Supplemental Fig. S1). Finally, Calpain-specific inhibition by Leupeptin (10 µM) added to the highest Calpain-2 concentration (10 U/ml) at least partially preserved the FL JP2 substrate, confirming the specificity of the proteolytic reaction cascade products (Fig. 1B). Importantly, we have mapped the analogous JP2 cleavage product cascade for Calpain-1 throughout the same neutral protease concentration range (Supplemental Fig. S2).

To identify specific Calpain-2 cleavage fragments, we developed a molecular weight-resolved mass spectrometry workflow. Using SDS-PAGE separation followed by data-dependent acquisition LC-MS/MS (DDA-MS) of Calpain-2 treated JP2 preparations and quantitation with a thresholded spectral counting approach, we identified abundant JP2 cleavage fragments already at the second lowest Calpain-2 concentration (0.1 U/ml). In combination with in silico cleavage prediction using DeepCalpain^25^, this enabled us to infer the positions of the corresponding cleavage sites (Fig. 1C,D, Supplemental Table S1). Accordingly, the first Calpain-2 cleavage site is localized between R557 (the last detected residue of NT_1_) and T566 (the first detected residue of CT_1_), while DeepCalpain confirmed R565 as the cleavage site reported previously^23^ (Fig. 1C top). The second cleavage site specifically generating NT_2_ is captured by DDA-MS within the tryptic peptide JP2^161-167^, because JP2^161-167^ (while detected for JP2 FL) disappears after the cleavage event, located between NT_2_ and NT_1_-NT_2_ (Fig. 1C middle). Here, DeepCalpain indicates S164 as the specific cleavage site. DDA-MS allocates the position of the third cleavage site that generates NT_3_ in the JP2 region between a.a. 236-295 (Fig. 1C bottom), where DeepCalpain predicts three potential cleavage sites: K243, S247, and G257. Finally, for the fourth cleavage site generating CT_2_, DDA-MS did not detect the corresponding cleavage products.

**Figure 2:**
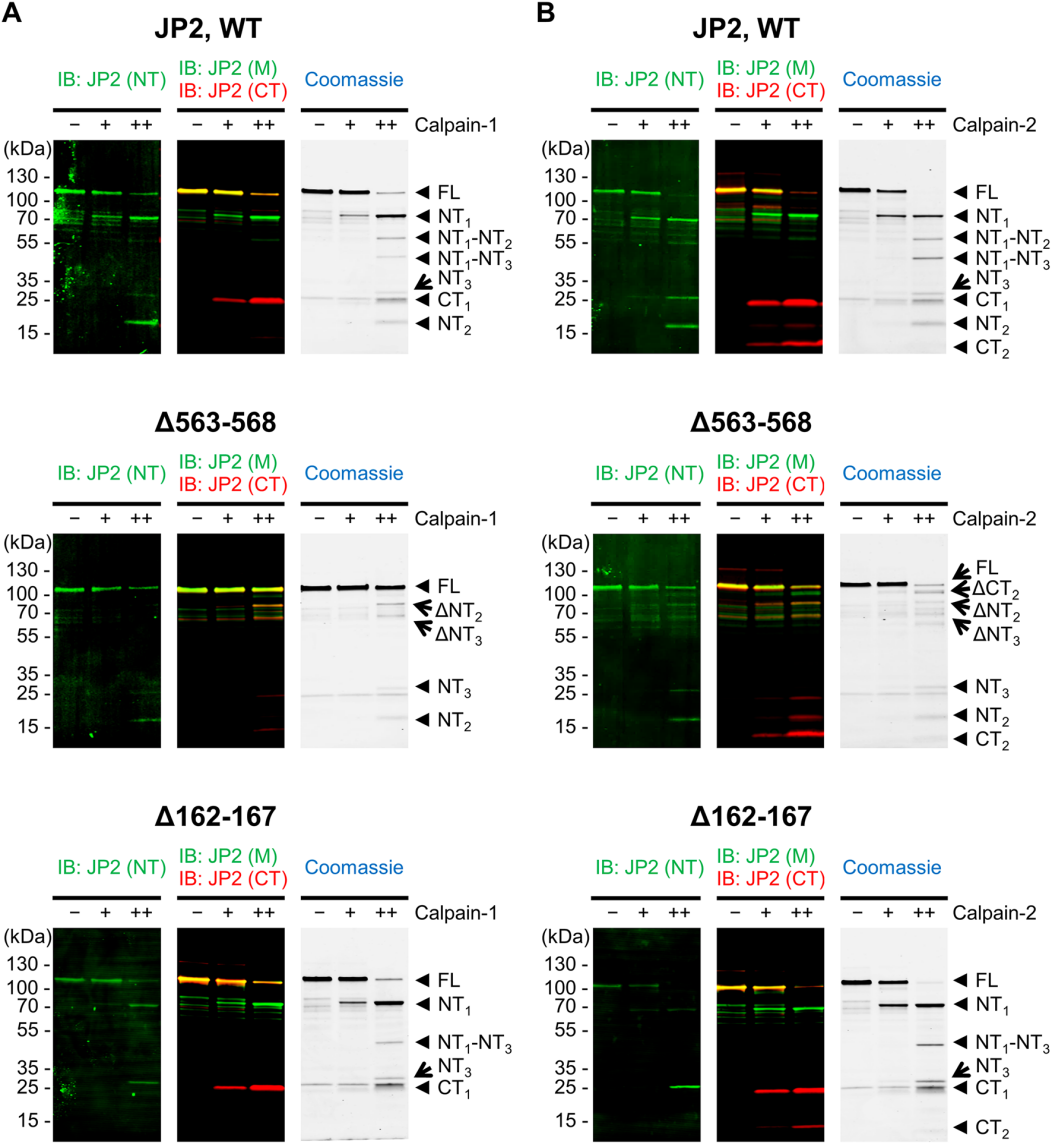
Proof-of-concept study of the primary and secondary Calpain-1 and Calpain-2 cleavage sites R565 and S164, respectively. Mouse JP2^WT^ versus the site-specific deletion mutants JP2^Δ563-568^ or JP2^Δ162-167^ were digested with 0.01 U/ml (+) or 0.10 U/ml (++) Calpain-1 (**A**) and Calpain-2 (**B**) for 30 min at 30 °C, and subjected to SDS-PAGE. The Calpain-specific cleavage patterns were detected by Coomassie staining and immunoblotting based on JP2 antibodies against the N-terminal (NT), middle (M) and C-terminal region (CT) indicated in Fig. 1A. Compared to WT (top), the JP2 deletion mutation Δ563-568 (middle) significantly changes the cleavage pattern by preventing the primary cleavage event. Importantly, the NT_1_ and CT_1_ fragments are not generated following Calpain-1 or Calpain-2 treatment of the JP2^Δ563-568^ deletion substrate. Additionally, the JP2 deletion mutation Δ162-167 (bottom) prevented the secondary cleavage event and the generation of the Calpain-1 and Calpain-2 specific NT_1_-NT_2_ and NT_2_ fragments.

Next, we confirmed the identity of the Calpain isoform-specific fragments by immunoblotting using epitope-specific antibodies (Supplemental Table S2). An N-terminal JP2 antibody raised against the JP2 epitope a.a. 66-115 detected both the fragments NT_2_ and NT_3_ following the proteolytic reaction both by Calpain-1 (Fig. 2A top) and Calpain-2 (Fig. 2B top) at the lower concentration (0.1 U/ml) also used for DDA-MS fragment identification. Of note, the N-terminal JP2 antibody showed only a weak signal for FL JP2 and NT_1_. Second, a JP2 antibody raised against the middle region of the human JP2 epitope a.a. 408-457 detected weak signals of the smaller fragments NT_1_-NT_3_ and NT_1_-NT_2_, but strong signals of the larger fragment NT_1_ and FL JP2. Third, an antibody raised against the human JP2 C-terminus epitope a.a. 431-680 detected a strong FL JP2 signal, as well as the fragments CT_1_ and CT_2_. Finally, the specificity of the C-terminal antibody binding was investigated by the epitope deletion mutation Δ644-649 of mouse JP2 (negative control), completely abolishing the C-terminal antibody binding and thus JP2^Δ644-649^ detection (Supplemental Fig. S3).

To confirm that Calpain-1 and Calpain-2 initially cleave JP2 at R565 and subsequently at S164, we purified and subjected the cleavage site deleted JP2^Δ162-167^ and JP2^Δ563-568^ proteins to Calpain-1 or Calpain-2 proteolysis at the lower concentration range (0.01 U/ml and 0.1 U/ml). In comparison to the WT FL JP2 substrate, the deletion mutation Δ563-568 of the JP2 substrate changed the Calpain-1 and Calpain-2 cleavage pattern. Specifically, the JP2 deletion at Δ563-568 prevented the generation of the fragments NT_1_ and CT_1_ following proteolysis by Calpain-1 (Fig. 2A middle left) and Calpain-2 (Fig. 2B middle right), thus identifying the JP2 region between a.a. 563-568 as the essential primary cleavage site. In agreement with absent primary cleavage, secondary cleavage of JP2^Δ563-568^ at the higher Calpain-2 concentration (0.1 U/ml) generated the cleavage fragment pairs i) NT_2_ and ΔNT_2_, ii) NT_3_ and ΔNT_3_, and iii) CT_2_ and ΔCT_2_, but not the otherwise observed NT_1_ downstream cleavage products NT_1_-NT_2_ and NT_1_-NT_3_ (Fig. 2B middle). Finally, while the JP2 deletion Δ162-167 did not affect the primary cleavage reaction, specifically the secondary cleavage event of NT_2_ was prevented, as evidenced by the loss of the NT_1_-NT_2_ and NT_2_ fragments following digestion by Calpain-1 (Fig. 2A bottom left) and Calpain-2 (Fig. 2B bottom right).

Recently G482 was proposed as an alternative primary JP2 cleavage site in HEK293 cells, overexpressing recombinant FL JP2 as Calpain substrate following exposure to increased extracellular Ca^2+^ concentrations^24^. To confirm G482 as a primary JP2 cleavage site, we purified the corresponding JP2^Δ479-486^ deletion protein as a cleavage site-dead and thus reaction-specific negative control substrate. However, JP2^Δ479-486^ was effectively cleaved by both Calpain-1 and Calpain-2, while the cleavage pattern was conserved similar to the WT JP2 fragmentation products (Supplemental Fig. S4). Hence, comparing the FL JP2 versus Δ479-486 deletion JP2 proteolytic patterns excludes G482 as a primary cleavage site of Calpain-1 and Calpain-2, confirming R565 as the major primary Calpain cleavage site. Furthermore, the JP2 deletion mutation Δ565-566 impaired the primary cleavage event stronger than Δ562-563, indicating that the scissile bond is rather formed by R565-T566 than by Y562-A563 (Supplemental Fig. S5).

While Calpain-1 versus Calpain-2 digestion of JP2 generated overall similar cleavage patterns, we observed an unexpected difference between the C-terminal cleavage products. Only the lower concentration of 0.1 U/ml Calpain-2 (Fig. 2B top right) but not Calpain-1 (Fig. 2A top left) generated the CT_2_ fragment. Interestingly, this Calpain-2 specific reaction generating ΔCT_2_ is reproducible with the primary cleavage site-dead JP2^Δ563-568^ substrate (Fig. 2B middle). Finally, higher concentrations of Calpain-1 (Supplemental Fig. S2) and Calpain-2 (Supplemental Fig. S1) generate the C-terminal fragments CT_1_ and CT_2_, which were however more stable in the presence of Calpain-1. In summary, while both Calpain isoforms generate similar cleavage products with similar efficiencies, only Calpain-2 generates the CT_2_ fragment at the second lower concentration.

**Figure 3:**
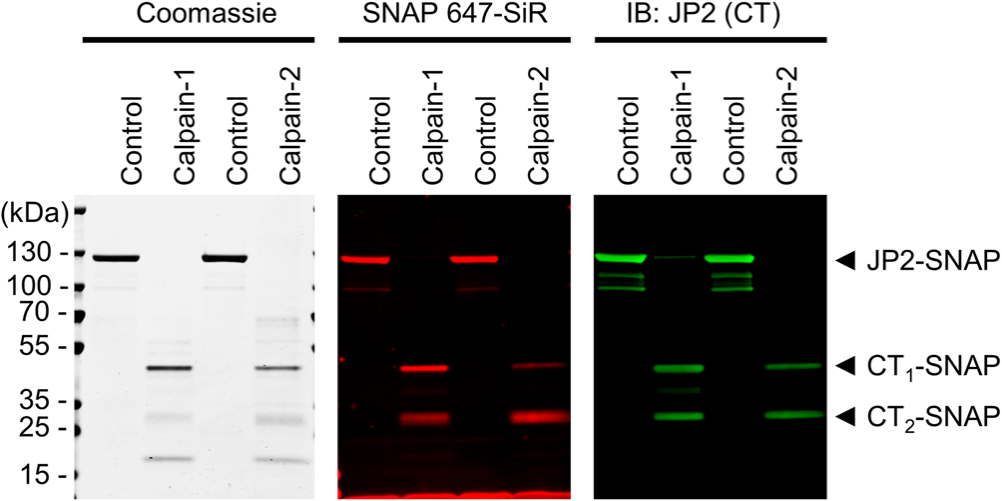
Identification of two unique C-terminal mouse FL JP2 cleavage fragments by SNAP-tagging and in-gel flourescent product detection. Recombinantly expressed, puried C-terminally SNAP-tagged JP2 was labeled with the high-affinity SNAP ligand 647-SiR and digested with 1.0 U/ml Calpain-1 or 1.0 U/ml Calpain-2 for 30 min at 30 °C. Digested samples were subjected to SDS-PAGE, Coomassie staining, in-gel 647-SiR flourescence detection, and immunoblotting as indicated. Two unique C-terminal fragments were identified by 647-SiR in-gel fluorescence, and each confirmed by Coomassie staining and immunoblotting, namely 647-SiR-tagged CT_1_ and CT_2_.

Next, based on covalent C-terminal JP2 SNAP-tag labeling, we analyzed the Calpain-dependent C-terminal cleavage reaction directly, using the fluorophore-labeled JP2-SNAP-647-SiR as substrate for in-gel fluorescence fragment detection. At the higher concentration of 1 U/ml both Calpain-1 and Calpain-2 generate the C-terminal CT_1_ and CT_2_ cleavage fragments (Fig. 3 SNAP 647-SiR). These data are further consistent with the conventional indirect immunoblotting results based on the C-terminal antibody confirming the CT_1_ and CT_2_ fragments (Fig. 3 IB: JP2 CT). Furthermore, because the SNAP-tag adds ~20 kDa to the C-terminal fragments, the apparent molecular weight shift of any hypothetical fragment with a molecular weight below CT_2_ would be identified by the covalent JP2 labeling assay. In summary, covalent 647-SiR labeling and immunoblotting prove that the C-terminal JP2 antibody detects the identical CT_1_ and CT_2_ cleavage fragments as the Calpain-1 and Calpain-2 specific products.

To confirm that native JP2 is cleaved in analogy in isolated mouse ventricular cardiomyocytes, we digested whole cell lysates each with Calpain-1 or Calpain-2 under the same reaction conditions followed by immunoblotting with JP2 antibodies against the middle and C-terminal JP2 epitopes. Apparently, the primary cleavage reaction of native JP2 generates the NT_1_ and CT_1_ fragments at the lower Calpain-1 or Calpain-2 concentration 0.1 U/ml, confirming the endogenous substrate reaction in murine adult cardiomyocytes (Supplemental Fig. S6). Moreover, endogenous FL JP2 is completely digested at the higher Calpain-1 and Calpain-2 concentration 1 U/ml, confirming that native JP2 is completely processed into the NT_1_ and CT_1_ fragments (Supplemental Fig. S6). Calpain-1 generated the secondary CT_2_, but no other cleavage products were observed at the highest concentration 10 U/ml, presumably because of antibody affinity limitations (Supplemental Fig. S6). As shown for the Calpain proteolysis of recombinant JP2, the JP2 M antibody fails to detect robustly the smaller cleavage products NT_1_-NT_2_ and NT_1_-NT_3_ (Fig. 2 top). Relative to the in vitro cleavage of purified JP2, higher Calpain concentrations were necessary to cleave native JP2 in cardiomyocyte lysates (Supplemental Fig. S6). While JP2, but not Caveolin3, RyR2 or GAPDH, was digested at the lower Calpain concentration 0.1 U/ml, GAPDH and RyR2 were effectively digested at the highest Calpain concentration 10 U/ml (Supplemental Fig. S6). In summary, exogenous Calpain proteolysis of native JP2 reproduces the substrate-specific reactions in murine cardiomyocytes, where JP2 is more susceptible to exogenous proteolysis compared to the RyR2 Ca^2+^ release channel.

### Identification of a C-terminal PEST motif overlapping with the primary Calpain cleavage site

Proline (P)-, glutamic acid (E)-, serine (S)-, and threonine (T)-rich sequences provide PEST recognition motifs relevant for proteolysis of short-lived proteins^26^. Interestingly, Calpain may also recognize its substrates through PEST motifs^27^. Analyzing the sequence of mouse JP2 with the PESTFind algorithm, we identified a previously unknown PEST motif in the C-terminal JP2 sequence, overlapping with the primary Calpain cleavage site R565 (Supplemental Table. S3, Supplemental Fig. S7B). The PESTFind algorithm evaluates the PEST motif likelihood by assigning a score ranging from -50 to +50, with positive values denoting potential PEST motifs. PEST scores above 5 are considered of particular interest and indicate target motifs^27^. This R565-associated PEST motif comprises 23 a.a. residues between positions 565-589 predicted with a high score of 26.9. Additionally, close to the primary R565 Calpain cleavage site we identified a second putative PEST motif comprised of 21 a.a. residues between positions 590-612, again predicted with a high score of 17.5, which may mediate the Calpain cleavage of the C-terminal fragment CT_2_. Since both C-terminal PEST motifs are located in the divergent region of JP2, we further confirmed the high degree of predicted intrinsic disorder by the IUPred^28^, PONDR-VLXT^29^, and DisEMBL^30^ algorithms (Supplemental Fig. 7S1A).

Intrinsically disordered domains have been described to increase the complexity and specificity of protein-protein interactions through unique disorder-to-order folding transitions^31,32^. Hence, we hypothesize that the divergent JP2 region facilitates its Calpain-specific substrate recognition through the two PEST motifs. Finally, two additional PEST motifs in proximity to the N-terminal Calpain cleavage sites are predicted, however, with relatively low scores (between positions 170-194 scored 3.36; or between positions 243-290 scored 7.52).

### Cytosolic Ca2+ increases nuclear NT1 translocation and local spot formation in living ventricular cardiomyocytes

**Figure 4:**
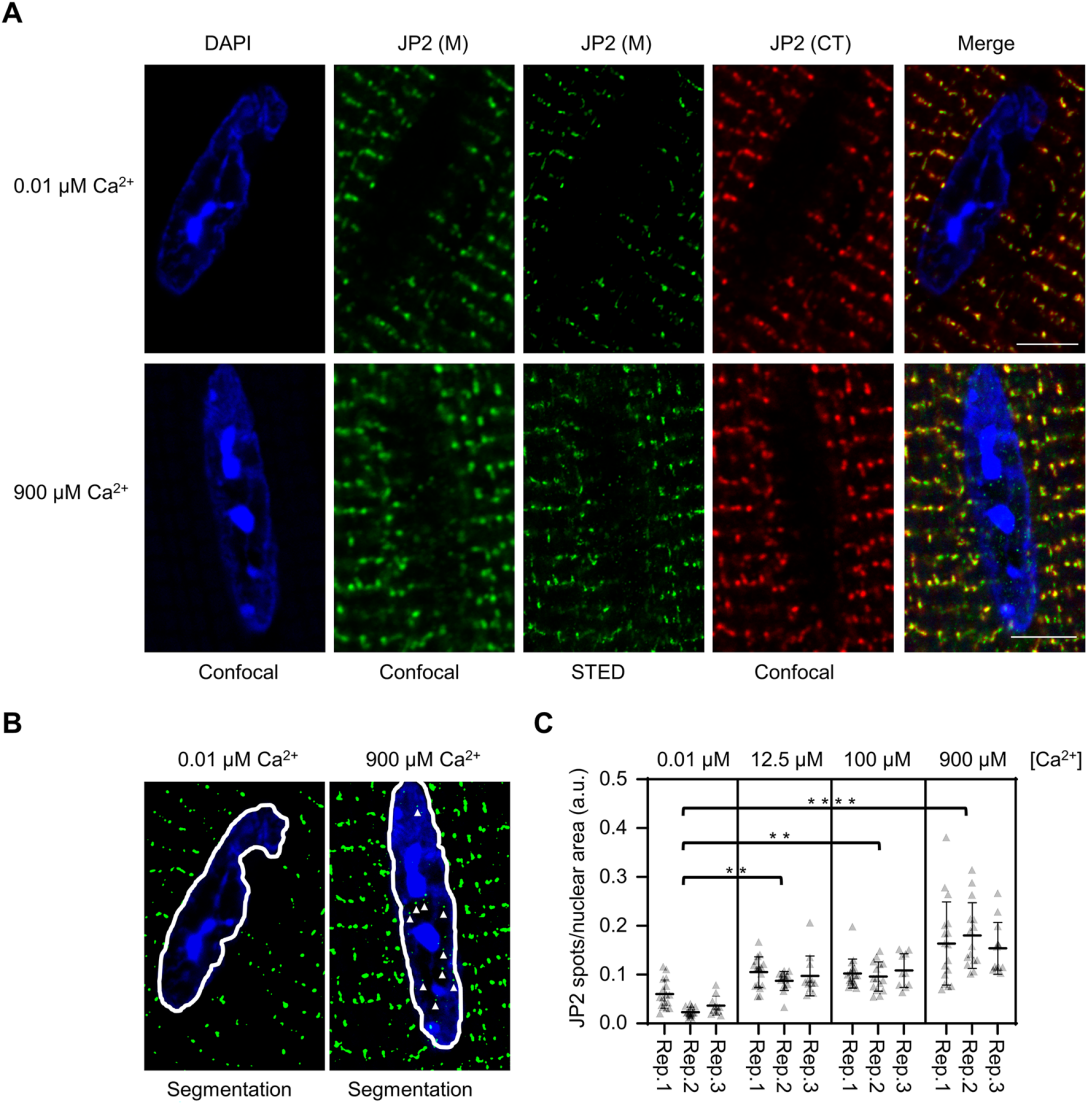
Micromolar extracellular Ca^2+^ concentrations increase the number of intranuclear JP2 signal spots in living ionomycin-permeabilized mouse cardiomyocytes. (**A**) Confocal and STED images showing DAPI-stained intranuclear DNA-rich regions and JP2 co-immunofluorescence M and CT signals. Following the ionomycin (2 µM) induced exposure to a 900 µM high extracellular Ca^2+^ concentration for 2 h, the antibody against the middle (M) JP2 portion robustly detected an increased number of small intranuclear JP2 signal spots, each visualized (green) by confocal and confirmed by STED imaging (compare 0.01 µM versus 900 µM). However, confocal imaging with the C-terminal JP2 antibody (CT) excluded any intranuclear signals (red). As expected, both the JP2 M and CT epitope-specific antibodies robustly detected the large cytosolic cluster signals with the typical striated pattern in cardiomyocytes (positive control). Scale bars, 5 μm. (**B**) Representative STED image segmentation examples for counting of the intranuclear number of JP2 signal spots labeled with the antibody against the M epitope (green). White triangles identify very small intranuclear JP2 signal spots in proximity but not colocalized with DNA-rich densely DAPI-labeled signal regions in central nuclear imaging planes suggesting not hetero- but euchromatic localization of intranuclear JP2. Apparently, the number of intranuclear JP2 signal spots is increased after exposure to the 900 µM extracellular Ca^2+^ concentration. Same magnification as **A**. (**C**) Dot plot quantifiying intranuclear JP2 signal spots normalized to the intranuclear STED imaging area in living cardiomyocytes exposed to increasing extracellular Ca^2+^ concentrations at 37 °C. Cultured ventricular cardiomyocytes were permeabilized with 2 µM ionomycin in culture for 2 h at 37 °C. Each Ca^2+^ concentration was analyzed in triplicate based on 3 biological replicates and at least 15 cardiomyocytes per group (please refer to the methods section for details). Data are presented as mean ± SD. Differences between groups were assessed by nested one-way ANOVA. **p < 0.01; ****p< 0.0001.

To analyze if endogenous JP2 cleavage products translocate to the nucleus as previously suggested^10,24^, we exposed ionomycin-permeabilized living ventricular mouse cardiomyocytes to increasing extracellular Ca^2+^ concentrations to activate their cytosolic Calpain isoforms. First, immunostaining against the JP2 middle and C-terminal regions confirmed the typical robust cytosolic JP2 signal localizations in transversally striated signals near the Z-disks but not inside the nucleus at the lowest Ca^2+^ concentration (Fig. 4A, 0.01 μM Ca^2+^). In contrast, confocal and superresolution STED imaging after exposure to the highest Ca^2+^ concentration revealed that the JP2 (M) but not the JP2 (CT) signal was translocated into the nucleus apparent as very discrete, small punctate local JP2 signal spots (Fig. 44A, 900 μM Ca^2+^).

Based on a nominal ~50 nm lateral STED resolution and established image segmentation workflows^21,33,34^, the intranuclear JP2 (M) signal spots and the DAPI labeled dense heterochromatin typically did not overlap, but rather showed a close nanometric association (Fig. 4B). Thus, the intranuclear JP2 preferentially localizes in areas of loosely packed chromatin where the transcriptionally active euchromatin is typically found^35^. Importantly, every additional Ca^2^^+^ concentration step increased the number of intranuclear JP2 (M) spots significantly in permeabilized cardiomyocytes isolated from different mouse hearts (Fig. 4C). Thus, endogenous WT JP2 (M) was cleaved by Calpain in a Ca^2+^ concentration-dependent manner and translocated to the nucleus in living cardiomyocytes following exogenous activation, confirming the physiological activity of the nuclear import for the post-cleavage specific fragment NT_1_.

Furthermore, consistent with NT_1_ translocation following local membrane-associated Calpain cleavage, the highly abundant, clustered cytosolic striated JP2 signals are retained, whereas the highly localized nuclear NT_1_ spots are represented by numbers of a much smaller JP2 signal subclass. In summary, based on DAPI staining and nuclear STED image analysis we exclude a significant signal overlap, while the in situ data suggest a close spatial association between euchromatic regions and highly localized NT_1_ signal spots.

### Human iPSC-derived cardiomyocytes generate a nuclear JP2 fragment

**Figure 5:**
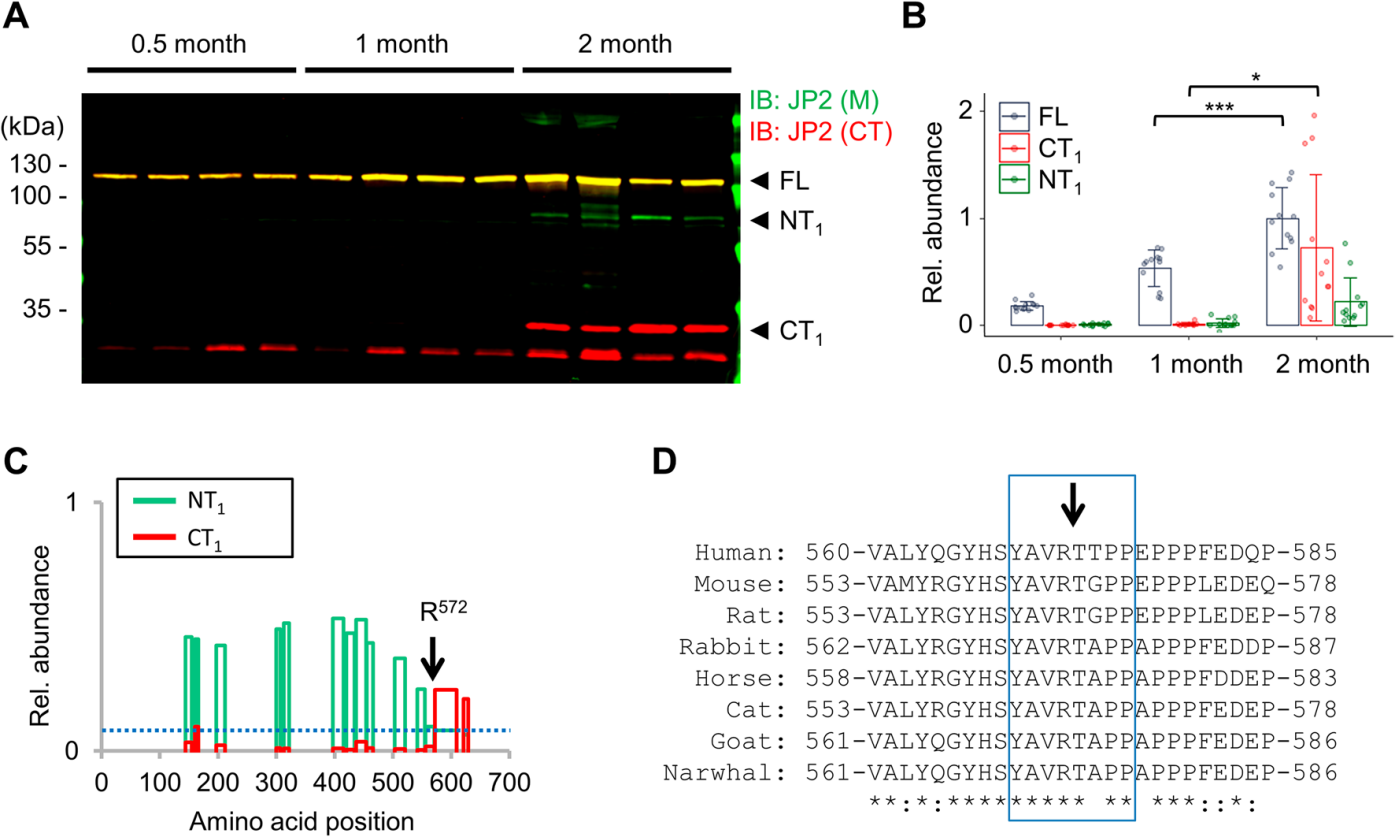
Calpain-specific cleavage fragments of JP2 are significantly increased in hiPSC-CMs after 2 months maturation, but not after 0.5 or 1 month differentiation. (**A**) Representative Western blot showing the protein levels of FL JP2 versus the Calpain-specific cleavage fragments. Both NT_1_ and CT_1_ were detected only after 2 months maturation, in contrast to the shorter 0.5 and 1 month hiPSC-CM differentiation time points. (**B**) Dot/bar plot quantifiying the Western blot data presented as mean ± SD (n = 4 biological replicates). Student’s t-test; *p < 0.05; ***p < 0.001. (**C**) Targeted data-dependent LC-MS/MS analysis of human Calpain-specific JP2 cleavage products in 2 months matured hiPSC-CMs. The relative abundance of the tryptic peptide peak areas of the endogenous human Calpain cleavage fragments is plotted versus the JP2 amino acid position including the primary human JP2 cleavage site R572 as indicated. (**D**) Sequence alignment confirming the high conservation of the primary Calpain cleavage site of JP2 across mammalian species from human to narwhal. Sequences human: NP_065166.2 (Homo sapiens); mouse: NP_001192005.1 (Mus musculus); rat: NP_001033063.1 (Rattus norvegicus); rabbit: NP_001075467.1 (Oryctolagus cuniculus); horse: XP_023482375.1 (Equus caballus); cat: XP_023106984.1 (Felis catus); goat: XP_017913386.1 (Capra hircus); Narwhal: XP_029077086.1 (Monodon monoceros). Asterisks mark identical residues; colons indicate highly conserved residues.

To investigate the JP2 expression and cleavage in human cardiomyocytes, we differentiate human iPSCs into ventricular cardiomyocytes as previously described^36^. Following 14 days differentiation of human iPSCs into cardiomyocytes (hiPSC-CMs), FL JP2 was robustly expressed, further increasing after 1 month and 2 months maturation (Fig. 5A). However, only after 2 months hiPSC-CM maturation, additionally the NT_1_ and CT_1_ Calpain-specific fragments were significantly cleaved (Fig. 5A,B). Using a highly specific molecular weight-resolved parallel reaction monitoring mass spectrometry (PRM-MS) assay for tryptic human JP2 peptides, we confirmed the identities of the human NT_1_ and CT_1_ cleavage products in hiPSC-CM lysates (Fig. 5C). In 2-month old hiPSC-CMs, the Calpain-specific cleavage site was identified between R572 (the last detected residue of NT_1_) and T573 (the first detected residue of CT_1_). Since PRM-MS did not detect any secondary Calpain cleavage fragments, these data indicate that only the primary Calpain cleavage activity occurs in hiPSC-CMs after 2 months maturation.

We compared the JP2 genetic protein sequence across eight mammalian species, confirming that the proposed primary Calpain cleavage site is highly conserved. Specifically, the human primary cleavage site (569-YAVR↓TTPP-576) differs from the mouse motif in only one residue: human T574 versus mouse G567 (Fig. 5D). Confocal immunofluorescence imaging showed a relatively low frequency of local overlap between JP2 in transversal striated signals at Z-disks and Calpain-1 signals in untreated 2 months mature hiPSC-CMs (Supplemental Figs. S8A,B). In contrast, a relatively high frequency of striated JP2 signals in proximity to the more abundant Calpain-2 signals was apparent (Supplemental Figs. S8C,D). These data confirm that Calpain-2 and JP2 are frequently localized in proximity in hiPSC-CMs after 2 months maturation.

**Figure 6:**
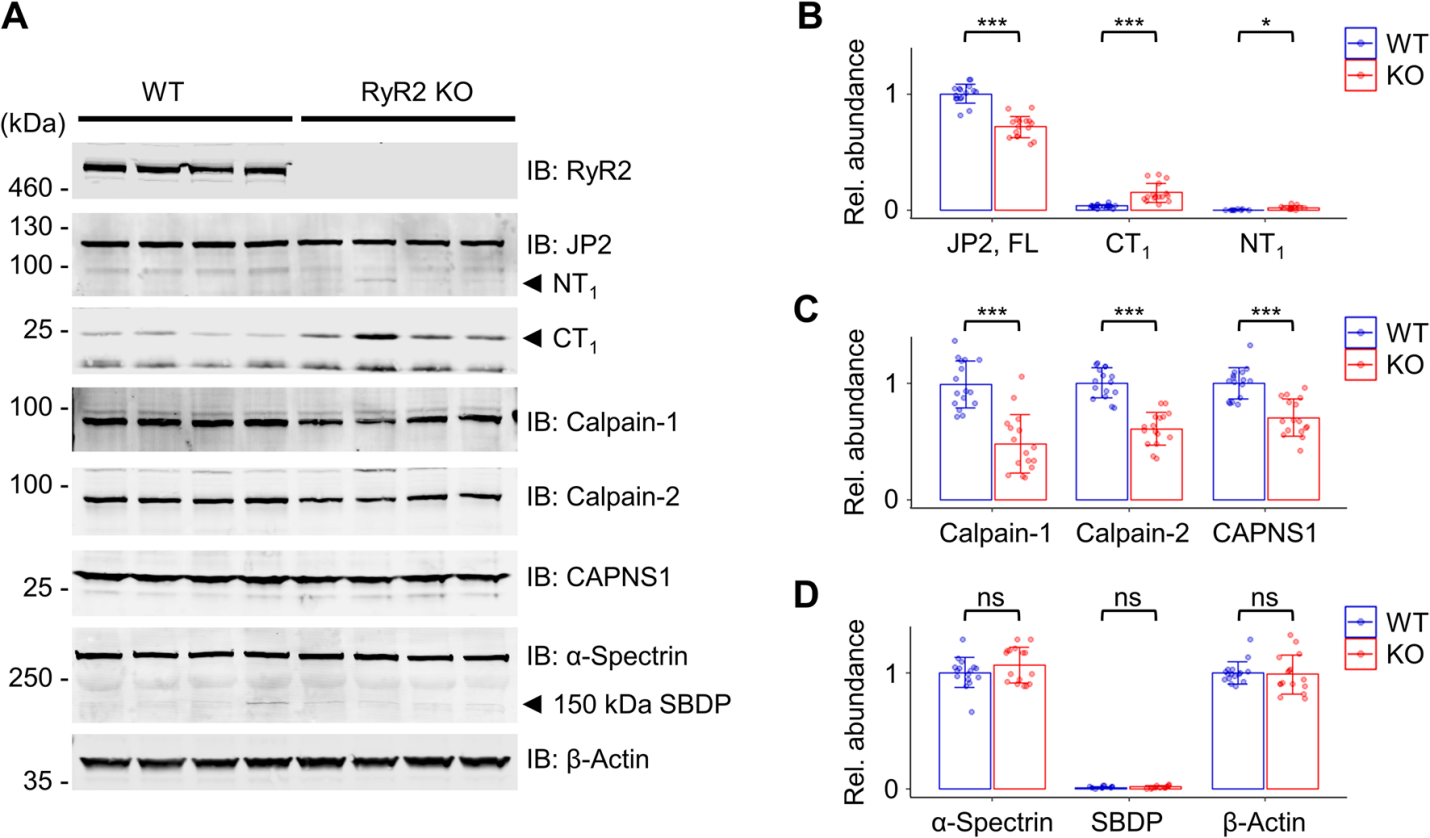
RyR2 knockout reveals an increased Calpain-specific fragmentation of human JP2 in 2 months matured hiPSC-CMs. (**A**) Western blots of WT versus RyR2 knockout hiPSC-CM lysates detecting the proteins RyR2, JP2 versus its Calpain-specific cleavage fragments NT_1_ and CT_1_, Calpain-1, Calpain-2, CAPNS1, and the Calpain-specific substrate α-Spectrin versus its 150 kDa α-Spectrin cleavage product SBDP. The complete absence of RyR2-specific band signals confirmed its knockout in hiPSC-CMs. β-Actin was used as loading control. (**B-D**) Dot/bar plots summarizing the immunoblot data of WT versus RyR2 knockout hiPSC-CM lysates. (**B**) In contrast to WT hiPSC-CMs, in RyR2 knockout hiPSC-CMs the relative abundance of FL JP2 was significantly decreased, whereas the abundance of its NT_1_ and CT_1_ fragments was significantly increased. (**C**) Calpain-1, Calpain-2, and CAPNS1 showed significantly decreased levels in RyR2 knockout iPSC-CMs. (**D**) The abundance of the Calpain-specific substrate α-Spectrin and its breakdown product SBDP were not significantly changed, confirming human FL JP2 as the specific substrate of the increased Calpain activity in RyR2 KO hiPSC-CMs. Data were normalized to REVERT total protein stain (please refer to the methods section for additional details). Data are presented as mean ± SD (n = 4 biological replicates). Student’s t-test; *p < 0.05; ***p < 0.001.

Since JP2 is very important for the maintenance of the cardiac dyad structure, we wondered how loss-of-function of RyR2 affects JP2 expression and cleavage. Therefore, we applied RyR2 knockout iPSC lines that were generated using the CRISPR/Cas9 gene editing technology^37^. In RyR2 knockout hiPSC-CMs, CICR is completely abolished, with accompanying impaired contractility and cell survival^37^. Here, we confirmed in 2 months matured RyR2 knockout hiPSC-CM lysates that tetrameric channel protomer expression is completely diminished (Fig. 6A). Strikingly, RyR2 deficient hiPSC-CMs generate the Calpain-specific cleavage fragments NT_1_ and CT_1_ at the cost of FL JP2 (Fig. 6A,B). Interestingly, this RyR2 knockout associated JP2 cleavage activity was accompanied by significantly decreased levels of Calpain-1, Calpain-2 and the Calpain small subunit 1 (Fig. 6C). However, the cytoskeletal cardiomyocyte protein α-Spectrin, a known Calpain-specific substrate^38^, and its cleavage product SBDP as well as β-actin were not targeted by the increased proteolytic activity in RyR2 knockout hiPSC-CMs (Fig. 6D). Thus, RyR2 knockout in hiPSC-CMs increases the proteolytic Calpain activity locally to cleave JP2, consistent with the previously reported JP2/RyR2 protein interaction^16^, shielding FL JP2 from the increased Calpain cleavage activity.

**Figure 7:**
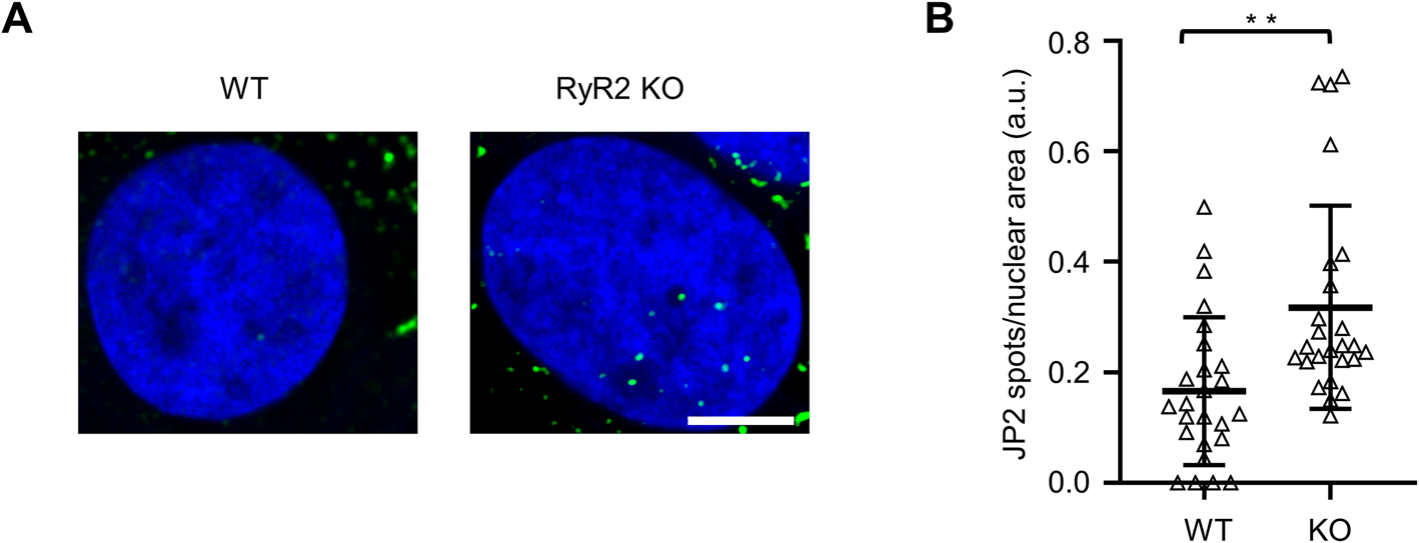
RyR2 knockout increases the number of discrete intranuclear JP2 signal spots in 2 months matured hiPSC-CMs. (**A**) Confocal images showing the DAPI-stained central nuclear imaging sections of WT versus RyR2 knockout hiPSC-CMs. Whereas one JP2 M epitope-labeled signal spot was apparent inside the WT hiPSC-CM nucleus, the number of signal spots was increased in RyR2 knockout hiPSC-CMs. Scale bar, 5 μm. (**B**) Dot plot comparing the intranuclear area-normalized number of JP2 signal spots between WT and RyR2 KO hiPSC-CMs. Data are represented as mean ± SD; n = 25 cells per group. Student’s t-test; **p < 0.01.

Consequently, we wondered if the human Calpain-specific JP2 cleavage product NT_1_ is also translocated into the nucleus in RyR2 knockout hiPSC-CMs. Using confocal immunofluorescence imaging for sensitive local signal detection, rarely detected discrete punctate intranuclear JP2 (M) signal spots near but not overlapping with DAPI labeled chromatin signals in hiPSC-CMs (Fig. 7A WT). In contrast, a strong increase in intranuclear JP2 (M) signal spots was apparent in RyR2 knockout hiPSC-CMs (Fig. 7A). Quantitatively, indeed the number of JP2 (M) spots per nuclear area section was significantly increased in RyR2 knockout hiPSC-CMs (Fig. 7B). Taken together, confocal intranuclear JP2 (M) imaging confirms the nuclear import of the Calpain-specific cleavage fragment NT_1_ in RyR2 knockout 2-month matured hiPSC-CMs with increased cleavage activity.

## Discussion

This study systematically investigated the previously unknown cascade of JP2 cleavage events through Calpain-1 versus Calpain-2 substrate-specific primary and secondary proteolysis steps, based on a multi-level proteomic and cardiac cell biology strategy that resulted in several major findings. First, our unbiased proteomic strategy revealed that both Calpain isoforms preferentially cleave mouse JP2 at R565. Second, we identified previously unknown Calpain-specific cleavage fragments and cleavage sites of JP2 that occur additionally after cleavage at R565. Third, the primary Calpain cleavage site is highly conserved in mammals. For the first time, we showed that the primary Calpain cleavage products NT_1_ and CT_1_ occur not only in mouse but also in human iPSC-derived cardiomyocytes. Fourth, RyR2 knockout in hiPSC-CMs destabilized FL JP2 generating the Calpain-specific cleavage fragments NT_1_ and CT_1_. This argues for a protective role of RyR2 against Calpain cleavage of JP2. Finally, we directly observed the translocation of the primary N-terminal cleavage product NT_1_ into the nucleus of mouse and human cardiomyocytes, where it closely associates with euchromatin-containing chromosomal regions. This underpins the molecular steps that underlie the proposed NT_1_ function as a transcriptional repressor reported previously to be involved in the cardio-protective regulation of reactive gene networks in heart failure^10^. Together these data support a model where the Calpain-specific proteolysis of JP2 generates a spectrum of cleavage products that may have altered biological functions, depending on the specific local Calpain isoform activities and the JP2 steric accessibility under different (patho-)physiological conditions. To gain a better functional understanding of the spectrum of Calpain-specific JP2 fragments, the precise knowledge of their molecular entities seems to be a key prerequisite for future studies.

Using purified recombinant mouse FL JP2, we established the complete molecular maps of the Calpain isoform-specific cleavage cascades *in vitro* under carefully controlled JP2 substrate-reaction conditions, throughout four orders of magnitude increasing enzyme concentrations from 0.01 to 10 U/ml. This revealed that both Calpain isoforms initially cleave the purified mouse JP2 substrate protein specifically at the primary cleavage site R565, which is located in the divergent region (Fig. 1A). Whereas the primary N- and C-terminal cleavage products NT_1_ (JP2^1-565^) and CT_1_ (JP2^566-696^) remain stable at the lowest Calpain-1 or Calpain-2 concentration (0.01 U/ml), a 10-fold higher Calpain concentration, while still in the lower range (0.1 U/ml), already resulted in three additional secondary cleavage events. Here, we identified S164 as a novel secondary cleavage site in the JP2 joining region generating the secondary N-terminal fragment NT_2_ (JP2^1-164^) and the large central fragment NT_1_-NT_2_ (JP2^165-565^). The joining region is subject to an additional secondary cleavage event at a.a. ~250 (potential cleavage sites: K243, S247, or G257) generating the fragments NT_3_ and NT_1_-NT_3_.

The C-terminal secondary cleavage event occurs in the divergent region. Thus, the joining region and the divergent region are the hotspots for the Calpain proteolysis. The joining region is functionally important, linking the MORN1-6 and MORN7-8 lipid binding domain clusters through a long cytosolic loop. Interestingly, an interaction in cultured cat ventricular cardiomyocytes between an adenovirally overexpressed HA-tagged human FL JP2 fusion protein and the native voltage-gated cardiac L-type Ca^2+^ channel Ca_V_1.2 α1C pore but not its auxiliary β2a subunit has been demonstrated by immunoprecipitation recently^39^. Importantly, when a human HA-JP2 fusion protein with seven random missense mutations in the joining region was overexpressed in feline cardiomyocytes, a weaker interaction with the native Ca_V_1.2 α1C protein was observed^39^. Thus, we speculate that the secondary Calpain cleavage site at S164 or at a.a. ~250 in the JP2 joining region may additionally disrupt the physiologically important interaction with the Ca_V_1.2 α1C channel and its localization in junctional membrane contacts, membrane-protein complexes that provide the local nanodomain control for elemental subcellular Ca^2+^ release events (i.e., Ca^2+^ sparks)^15^.

Interestingly, bioinformatic mouse JP2 sequence analysis (Fig. S7B) showed that the primary R565 cleavage site is closely associated with two putative PEST motifs with the highest predicted scores (a.a. 565-589 and 590-612). PEST motifs are generally known to and may thus facilitate a more rapid JP2 cleavage through Calpain-specific substrate recognition and digestion^27^. While overall five PEST motifs were predicted for JP2, only the three C-terminal PEST motifs that are located in the divergent region resulted in higher scores, with PEST4 directly overlapping or PEST5 closely associated with the primary Calpain cleavage site R565. The other two putative PEST motifs (PEST1 and PEST2) are located in the joining region of JP2. The most N-terminal putative PEST1 motif in proximity to the secondary N-terminal cleavage site S164 has a relatively low predicted score. The predicted motif PEST2 (a.a. 243-290), predicted with an intermediary score, is associated with the third Calpain cleavage site at a.a. ~250 (Fig. 1C). Finally, the putative PEST3 motif (a.a. 468-488) comprises the proposed Calpain-2 specific cleavage site at G482^24^. Henceforth, we confirmed the R565 cleavage site-specific primary Calpain cleavage fragments in ventricular mouse and human iPSC-derived cardiomyocytes.

### NT1 nuclear translocation results in local fragment compartmentation and spatial association with euchromatic chromosomal regions in cardiomyocytes

Recently, a nuclear translocation after Calpain cleavage was reported both for the N-terminal and C-terminal JP2 cleavage fragments based on confocal imaging^10,24^. Interestingly, Lahiri et al. reported that a monopartite nuclear localization signal (NLS) in the mouse JP2 divergent region (a.a. 484-492) is directly located behind the alternative C-terminal Calpain-2 specific cleavage site G482, generating the fragment JP2^483-696^ for subsequent nuclear translocation. However, in human and mouse cardiomyocytes we could not detect any JP2 cleavage fragments or intranuclear JP2 (CT) signal spots that correspond with the putative G482 cleavage site. Interestingly, a relatively diffuse intranuclear post-cleavage NT_1_ signal distribution has been reported in mouse ventricular cardiomyocytes previously^10^, whereas our corresponding confocal imaging data showed discrete local intranuclear JP2 (M) but not JP2 (CT) immunofluorescent signal spots, suggesting a highly compartmentalized local NT_1_ accumulation. We demonstrated highly localized NT_1_ signals by STED superresolution imaging in close proximity but not overlapping with the densely DAPI-stained heterochromatic regions. This suggests that intranuclear NT_1_ preferentially associates with the loosely packed euchromatin which is typically transcriptionally active^35^. Hence, the proposed cardio-protective NT_1_ function as a transcriptional post-cleavage repressor of reactive gene networks in heart failure reported previously^10^ may depend on local N-terminal fragment-rich compartments associated with specific chromosomal regions.

Importantly, the primary Calpain cleavage site at R565 is highly conserved, particularly in the human and mouse JP2 protein investigated here. We identified the endogenous human and mouse primary Calpain cleavage fragments NT_1_ and CT_1_ in hiPSC-CMs and mouse ventricular cardiomyocytes consistently, using JP2 antibodies against the middle (M) versus C-terminal (CT) region. In WT hiPSC-CMs, NT_1_ and CT_1_ were detected following an increase in JP2 expression during 2 months maturation (Fig. 5A,B). This observation is consistent with previous studies reporting an increased expression of JP2 and other SR proteins in maturated hiPSC-CMs^40,41^. Interestingly, in the absence of any immunoblot detectable RyR2 protein in hiPSC-CM knockout cells, we identified a significantly decreased immunoblot level of FL JP2 compared to WT hiPSC-CMs (Fig. 6A,B), which accompanies the defective excitation-contraction (E-C) coupling in RyR2 knockout hiPSC-CMs shown previously^37^. Together, these findings are in line with previous studies showing that JP2 tethers the plasma membrane to the SR and is essential in establishing and maintaining functional cardiac dyads, the structural basis for E-C coupling in cardiomyocytes^11^. In parallel after 2 months RyR2 knockout hiPSC-CM maturation increased immunoblot levels of the Calpain-specific primary NT_1_ and CT_1_ cleavage products in RyR2 knockout hiPSC-CMs indicates a destabilization of the human FL JP2 protein. Finally, targeted mass spectrometric analysis by parallel reaction monitoring (PRM) revealed the spectrum of the endogenous NT_1_ and CT_1_ fragments in 2-month matured hiPSC-CM knockout cells, identifying R572 as the Calpain-specific human JP2 cleavage site.

The mouse JP2 protein is known to contain two nuclear localization signals (NLS): (1) the bipartite NLS (a.a. 345-359)^10^ and (2) the monopartite NLS (a.a. 488-492)^10,24^. Both NLS sequences are evolutionary conserved in human JP2 (bipartite NLS: a.a. 351-365; monopartite NLS: a.a. 495-499). Accordingly, the FL JP2 cleavage at the human R572 site generates a primary NT_1_ fragment containing two nuclear localization signals. Intranuclear JP2 (M) high-resolution confocal imaging showed a significantly increased post-cleavage import of the human NT_1_ fragment apparent as highly localized signal spots in hiPSC-CM (Fig. 7). Superresolution STED imaging demonstrated that exposure of living ionophor-permeabilized mouse ventricular cardiomyocytes to higher micromolar Ca^2+^ concentrations significantly increases the translocation of NT_1_ into the nucleus and thus the number of discrete intranuclear JP2 (M) signal spots. Vice versa, confocal JP2 (CT) immunofluorescence imaging excluded any intranuclear uptake of the CT_1_ fragment. Furthermore, we show for the first time a nanometric association of intranuclear JP2 (M) signal spots with weakly DAPI-stained euchromatin in mouse cardiomyocytes, while excluding overlapping co-localized DAPI-dense heterochromatin and JP2 (M) signal spots.

Previous studies have relied solely on confocal imaging, however, with limited lateral resolution (xy plane ~250–300 nm)^21^ to confirm the nuclear import of the NT_1_ fragment in mouse cardiomyocytes^10^. Here, we introduce superresolution JP2 (M) signal spot STED imaging and quantitative image segmentation workflows, leading to a new intranuclear post-translocation local NT_1_ compartment model: following intranuclear NT_1_ import the primary Calpain cleavage products accumulate in very small foci locally, spatially associated with transcriptionally active euchromatic regions, where JP2 is proposed to interact with TATA box motifs^10^. Future studies will need to identify the molecular mechanisms of the highly localized NT_1_ fragment accumulation and the functional role of the accumulation. Additionally, confocal imaging data in RyR2 knockout hiPSC-CM nuclei qualitatively extend a similar human NT_1_ translocation process, confirming the compartmentalized accumulation of the Calpain-specific cleavage product and the close spatial association with euchromatic chromosome regions. Interestingly, FL JP2 appears to be more susceptible to Calpain cleavage at a lower enzymatic activity (1 U/ml) compared to RyR2 (10 U/ml) in mouse ventricular cardiomyocyte lysates treated with Calpain-1 or Calpain-2 (Fig. S6), and in living permeabilized RyR2 knockout hiPSC-CM exposed to increasing extracellular Ca^2+^ concentrations (0.01-900 µM). Hence, RyR2 knockout in hiPSC-CMs uncovered protective and specific roles of RyR2 channels against Calpain-dependent JP2 cleavage presumably through protein-protein interactions. Indeed interactions between JP2 and RyR2 in cardiomyocytes have been identified previously^42^. The nuclear accumulation of NT_1_ in RyR2 knockout hiPSC-CMs may regulate genes involved in cardiomyocyte survival and prevent cell death, as RyR2 knockout hiPSC-CMs could survive in vitro more than 3 months^37^. In summary, further studies are warranted to investigate the JP2 NT_1_-targeted transcripts in hiPSC-CMs.

### Excluding putative JP2 cleavage sites establishes R565 as the primary Calpain target

Because several alternative primary Calpain-specific JP2 cleavage sites have been proposed, it is important to re-consider each L201^16^, G482^11^, and R565^23^ in the context of our DDA-MS datasets. This unbiased proteomic strategy demonstrated that both Calpain-1 and Calpain-2 cleave mouse JP2 primarily at amino acid position R565, however, not at L201 or G482. Different biochemical, bioinformatic, and data interpretation strategies may explain the earlier discrepant findings. For accurate assessment of the isoform-specific Calpain cleavage cascades, it is important to take the anomalous gel migration behavior of FL JP2 and its cleavage fragments into full consideration. FL JP2 has a calculated MW of ~74 kDa, however, under denaturing conditions in SDS-PAGE the protein migrates at ~100 kDa. Accordingly, the corresponding gel electrophoretic mobility deviates for the primary cleavage fragments NT_1_ (apparent MW ~75 kDa versus calculated MW 60 kDa) and CT_1_ (apparent MW ~25 kDa versus calculated MW 14 kDa), and less for the secondary NT_2_ fragment (apparent MW ~20 kDa versus calculated MW 17 kDa). In summary, the particularly anomalous gel migration of the fragment CT_1_ is reflected by a remarkably large gel shift factor of ~1.8 between the apparent versus the expected MW, which can lead to erroneous interpretation of Calpain cleavage sites solely based on the proteolytic fragments. Interestingly, the C-terminal region of JP2 is characterized by a relatively high proline content (FL JP2 9.8% versus CT_1_ 17.6%). It has been reported that proline-rich proteins migrate slower on SDS-PAGE, presumably because of their increased structural rigidity^43–45^. Together with the fact that glutamic acid, serine, and threonine, are enriched with proline in the JP2 sequence, these domains may function as PEST motifs. Bioinformatic sequence analysis revealed that JP2 contains a C-terminal PEST4 motif (a.a. 565-589) overlapping with the primary Calpain cleavage site R565. Specifically, the PEST4 motif starts with a highly conserved proline-rich sequence (566-TGPPEPPP-573) (Fig. 5D). A potentially important function of this PEST4 motif is supported by our findings, demonstrating that the domain-specific deletion mutation of the scissile bond Δ565-566 prevents only partially the Calpain-specific cleavage, whereas JP2 truncated at Δ563-568 prevented the cleavage nearly completely.

While proline residues tend to be excluded from α-helices and β-sheets, proline-rich sequences are frequently found in intrinsically disordered regions of proteins^46,47^. This is fully consistent with the high degree of intrinsic disorder predicted bioinformatically for the C-terminal divergent mouse JP2 region (a.a. 430-674). A high degree of intrinsic disorder is associated with more effective steric substrate accessibility and flexibility, general structural substrate characteristics facilitating cleavage of substrates by proteases^48^. Besides the degree of intrinsic disorder, proline-rich sequences can provide key structural elements directly involved in the protease-specific substrate recognition mechanism of Calpains^49^. Prolines frequently dominate the segment C-terminal to the scissile bond, where they occupy the positions P2’-P4’ of the Calpain cleavage site. This is in agreement with P568 and P569 occupying the Calpain-specific JP2 substrate positions P3’ and P4’. Moreover, the preferred residues of the scissile bond are lysine, tyrosine, and arginine in position P1 and serine, threonine, and alanine in P1’, in agreement with R565 identified as the P1 and T566 as P1’ sites^49^. Together with R565 at site P1, the hydrophobic residue V564 in P2 corresponds to the P2-P1 preference rule model, which requires P2 to be preferably occupied by leucine and valine residues^50,51^. Thus, the unique primary Calpain cleavage site R565 identified here through the JP2 fragmentation cascade mapping as the preferentially cleaved site fits very well to the amino acid preferences generally attributed for Calpain as a neutral protease.

However, it has also been suggested that the Calpain cleavage mechanism depends nonetheless to a large extent on higher order structural clues and less on sequence determinants resulting in less precise *in silico* predictions^50,52^. This notion is further consistent with the preferred Calpain substrate recognition based on the intrinsically disordered JP2 region located between the α-helical region and the transmembrane domain^4^.

Importantly, this primary cleavage site is highly conserved across mammalian species. Accordingly, we identified R572 in human JP2 as Calpain-specific primary cleavage site in hiPSC-CMs, implying functional conservation of the protease-substrate relationship between Calpain and JP2 during evolution.

### Study limitations and avenues for further research

A major study limitation is given by the lack of Calpain cleavage site-specific antibodies, materials that would allow to specifically track endogenous JP2 fragments in the presence of FL JP2. In particular, the subcellular localization and function of the primary C-terminal cleavage product CT_1_ remains unknown. Further studies are needed to delineate the Calpain-specific subcellular breakdown, sorting, and translocation pathways of endogenous JP2 in cardiomyocytes under (patho-)physiological conditions.

## Conclusions

Taken together, this study provides the first systematic molecular analysis of the JP2 cleavage products by Calpain-1 versus Calpain-2 proteolysis throughout four orders of increasing enzymatic activities, revealing that both Calpain isoforms preferentially cleave mouse JP2 at R565. This cleavage site is characterized by an evolutionary conserved sequence matching the general Calpain-specific amino acid preferences, overlapping with a potential PEST motif, and localized in the intrinsically disordered divergent region. Hence, these molecular determinants of this cleavage site R565 fit very well to the preferred substrate scheme generally attributed to Calpain-1 and Calpain-2.

Close association with potential PEST motifs is also observed for the three secondary cleavage sites, two of them are located in the joining region and one in the divergent region of JP2, qualifying these domains as the hotspots of the Calpain-specific cleavage.

(Patho-)Physiological relevance of the Calpain cleavage of JP2 is implicated by the nuclear translocation of the Calpain-specific primary cleavage product NT_1_, associating with small gene-rich euchromatic regions in adult mouse cardiomyocytes after exposure to Ca^2+^ and human iPSC-CMs after RyR2 knockout, where it is proposed to function as a cardio-protective transcriptional regulator in heart failure^10^. Hence, stabilizing NT_1_ by preventing secondary cleavage events by Calpain and other proteases could be an important therapeutic target for future studies.

## Methods

### Antibodies

The primary antibodies used in this study were against: JP2 (N-terminal region; rabbit polyclonal; Abcam, ab116077), JP2 (middle region; rabbit polyclonal; Fitzgerald, 70R-6923), JP2 (C-terminal region; mouse monoclonal; Santa Cruz, sc-377086), RyR2 (rabbit polyclonal; Sigma-Aldrich, HPA020028), Calpain-1 (mouse monoclonal; Santa Cruz, sc-271313), Calpain-2 (rabbit polyclonal; Santa Cruz, sc-30064), CAPNS1 (rabbit polyclonal; Sigma-Aldrich, HPA006872), α-Spectrin (rabbit polyclonal; Cell Signaling, #2122), and β-Actin (mouse monoclonal; Santa Cruz, sc-47778). The secondary antibodies used for Western blot analysis were: anti-mouse IRDye 680RD (donkey polyclonal; LI-COR, #926-68072), and anti-rabbit IRDye 800CW (donkey polyclonal; LI-COR, #926-32213). The secondary antibodies used for immunofluorescence were: anti-mouse-STAR635P (goat polyclonal; Abberior, 200020075), and anti-rabbit-STAR580 (goat polyclonal; Abberior, 200120058).

### Prediction of Calpain cleavage sites, PEST motifs, and intrinsically disordered regions in silico

In silico prediction of the Calpain cleavage sites and PEST motifs in the Junctophilin-2 protein sequence from mouse (NP_001192005.1) or human (NP_065166.2) was performed using the DeepCalpain tool^25^ and PESTfind tool^27^, respectively. Intrinsically disordered regions of mouse JP2 were predicted using IUPred^28^, PONDR-VLXT^29^, and DisEMBL^30^ algorithms.

### Protein expression and purification of recombinant JP2

Mouse Junctophilin-2 cDNA (NP_001192005.1) was amplified by PCR and cloned into pGEX-6P-1 vector (GE Healthcare). Site-directed mutagenesis of Δ162-167, Δ479-486, Δ563-568 and Δ644-649 was carried out using the GeneArt™ site-directed mutagenesis kit (Thermo Fisher Scientific). For specific dye labeling, the SNAP-tag sequence (New England BioLabs) was amplified by PCR and cloned into the vector immediately downstream of the JP2 sequence, while the stop codon of JP2 was removed by site-directed mutagenesis. Sequences were confirmed by DNA sequencing. Recombinant JP2 was expressed as N-terminally GST-tagged fusion protein in Escherichia coli BL21 induced by addition of 1.0 mM IPTG at OD 0.6 followed by incubation at 30 °C for 4 h. After cell lysis and centrifugation, the protein in the supernatant was affinity purified on Gluthatione Sepharose 4B prepacked columns (GE Healthcare). The tag was removed by on-column cleavage using PreScission Protease (GE Healthcare) resulting in the elution of untagged JP2. Finally, JP2 was purified by size-exclusion chromatography using a HiLoad 16/60 Superdex 200 column (GE Healthcare) in 25 mM Tris-HCl, pH 7.5, 150 mM NaCl, 2 mM DTT, 1% Triton X-100.

### In vitro cleavage by Calpain-1 or Calpain-2

Purified recombinant JP2 (0.1 nmol) or whole cell lysates of mouse ventricular myocytes (50 µg) were incubated for 30 minutes at 30 °C with Calpain-1 (Sigma-Aldrich; 0.01-10 U/ml) or Calpain-2 (Calbiochem; 0.01–10 U/ml) in 50 µl reaction buffer (25 mM Tris-HCl, pH 7.5, 150 mM NaCl, 2 mM DTT, 1.0 mM CaCl2, 0.5% Triton X-100). If SNAP-tagged JP2 was used as substrate, the SNAP-tag was labeled with SNAP ligand 647-SiR before the proteolytic reaction according to the manufacture instructions (New England BioLabs). For negative control, samples were preincubated with 10 µM Leupeptin for 10 min on ice. The proteolysis was terminated by the addition of Laemmli sample buffer, followed by boiling for 5 min. The cleavage products were resolved on SDS-PAGE and analyzed by Coomassie-staining, 647-SiR fluorescence, immunoblotting using JP2 antibodies against the N-terminal (Abcam), middle (Fitzgerald), and C-terminal region (Santa Cruz), or LC-MS/MS.

### Activation of intracellular Calpain by exposure to Ca2+/ionomycin

Isolated mouse ventricular myocytes or hiPSC-CMs were cultured on glass slides covered with laminin (BD Biosciences). Adherent cells were treated with 2 µM ionomycin (Sigma-Aldrich) and 0.1 nM to 900 µM free Ca^2+^ buffered with EGTA (Sigma-Aldrich) in HEPES buffer (20 mM HEPES, 150 mM NaCl, pH 7.4) for 2 h at 37 °C. Free Ca^2+^ concentrations were calculated using the WEBMAX-C software (C. Patton, Stanford University)^53^. Cells were fixed and permeabilized for immunofluorescence microscopy.

### hiPSC-derived cardiomyocytes

The study was approved by the Ethics Committee of the Technical University Dresden (stem cell protocol approval number EK 422092019), and carried out in accordance with the approved guidelines. In Dresden, hiPSC lines WT (iBM76.1) and RyR2 KO (CRISPR_A3)^37^ were cultured on Geltrex-coated (Thermo Fisher Scientific) 6-well plates (CELLSTAR, Greiner) in E8 medium (Thermo Fisher Scientific), and passaged every 4-to-6 days with a cell dissociation reagent (Versene solution, Thermo Fisher Scientific). 2 µM Thiazovivin was added to the culture for the first 24 h after passaging. Directed differentiation of hiPSCs into hiPSC-derived ventricular cardiomyocytes was performed via WNT signaling modulation, as described previously.^36^ Briefly, hiPSCs at 80%–90% confluence were transferred into differentiation medium (RPMI 1640 medium (Gibco) containing 0.5 mg/mL human recombinant albumin (Sigma-Aldrich) and 0.2 mg/mL L-ascorbic acid 2-phosphate (Sigma-Aldrich)) and were treated with 4 µM of a GSK-3α/β inhibitor (CHIR-99021, Merck Millipore) for 48 hours and then 5 μM of a PORCN inhibitor (IWP2, Merck Millipore) for 48 hours. After ~15 days, hiPSC-derived cardiomyocytes were replated at lower cell density (1:2 to 1:3) in Geltrex-coated 6 well plates (CELLSTAR, Greiner). Subsequentially, hiPSC-derived cardiomyocytes with a purity of 85-95% were cultured in cardio culture medium (RPMI 1640 medium (Gibco) containing 2% B27 (Gibco)) for further maturation.

### Ventricular myocyte isolation from adult mouse hearts

All animal procedures were approved by the institutional animal care and use committee of the University Medical Center Göttingen (ex vivo cardiomyocyte isolation from extracted rodent hearts approval number: 21/1/11) and in line with Directive 2010/63/EU of the European Parliament. Ventricular myocytes were isolated from 8 to 20 weeks old C57BL6/N mouse hearts as previously described.^54^ Mouse hearts were excised, mounted via the aorta to a cannula connected to a modified Langendorff perfusion setup, and perfused with a modified Ca^2+^ free Tyrode buffer (120.4 mM NaCl, 14.7 mM KCl, 0.6 mM Na_2_HPO_4_, 0.6 mM KH2PO4, 1.2 mM MgSO4, 10 mM HEPES, 4.6 mM NaHCO_3_, 30 mM Taurin, 10 mM 2,3-Butanedione monoxime, 5.5 mM Glucose, pH 7.4) at 37 °C. Hearts were then enzymatically digested by adding 2 mg/mL collagenase type II and 40 µM CaCl_2_ to the Tyrode perfusion buffer for 9 min at 37 °C. The ventricles were dissected. Finally, the isolated ventricular cardiomyocytes were transferred into Tyrode buffer containing 10% FBS (Sigma-Aldrich) to terminate the enzymatic digestion.

### Immunofluorescence confocal microscopy and superresolution STED nanoscopy

For immunocytochemical analysis, cells were plated on glass coverslips covered with laminin (BD Biosciences), fixed (Roti-Histofix 4%, Carl Roth) at RT for 30 min, permeabilized with 0.1% Triton-X100 (Carl Roth) in 1% BSA (Sigma-Aldrich) in PBS (Gibco) at RT for 20 min, and blocked with 1% BSA (Sigma-Aldrich) in PBS (Gibco) at 4 °C overnight. Cells were incubated with primary antibodies diluted in blocking solution (1% BSA in PBS) at 4 °C overnight, washed thrice with blocking solution, and finally incubated with secondary antibodies anti-mouse-STAR635P (Abberior Instruments) or anti-rabbit-STAR580 (Abberior Instruments) diluted 1:1000 in blocking solution at RT for 1 h. The fluorescently labeled coverslips were mounted onto microscope slides using Prolong Gold Antifade Mountant with DAPI (Thermo Fisher Scientific), dried on air protected from light, and sealed with nail polish. Images were collected by confocal microscopy or STED nanoscopy using a custom-built Abberior RESOLFT QUAD P microscope system. The optical device setup included an Olympus IX83 inverted microscope, equipped with a 100x 1.4 NA oil-immersion objective and an Abberior QUAD beam scanner, controlled by the software Abberior Inspector (version 16.1.6905). Three fluorescent imaging channels were used in confocal scanning mode, herein designated as blue (405 nm excitation, 422–467 nm detection), green (594 nm excitation, 605–625 nm detection) and red channel (640 nm excitation, 650–720 nm detection). All lasers were pulsed and regulated by an acousto-optic modulator. The red channel was also used in STED mode, supported by a 775 nm synchronized depletion laser, which was aligned at the start of each imaging session using 40 nm red fluorescent beads (Abberior Nanoparticle Set for Expert Line 595 & 775 nm). In STED mode, a time gating of 0.5–8 μs was used. The pinhole size was set to one Airy unit in all recordings. Reported pixel intensities are photon counts detected by avalanche photodiode detectors without further processing. Z-stacks were recorded using continuous autofocus, an operation mode used to mitigate sample drift by compensation of small stage movements along the optical axis. Adjustment of the STED laser power to maximize resolution and raw images processing in Fiji (https://imagej.net/Fiji) were carried out according to previously established workflows.^21,33^

### Western blot analysis

Ventricular cardiomyocytes isolated from mouse hearts or hiPSC-CMs were homogenized in ice-cold RIPA buffer using a Potter homogenizer (RW20 digital, IKA). The homogenates were centrifuged at 10,000 × g for 10 min at 4 °C to remove insoluble contents and the protein concentrations determined by bicinchoninic acid assay (Pierce BCA Protein Assay; Thermo Fisher Scientific). Samples were heated for 5 min at 96 °C in 1x Laemmli buffer. Equal amounts of protein samples were loaded and separated on 4–12% gradient SDS-PAGE gels (Bio-Rad) by electrophoresis. Proteins were transferred onto polyvinylidene difluoride membranes (0.45 mm, Immobilon-FL, Merck Millipore). Protein transfer onto membranes was visualized by REVERT total protein staining (LI-COR). Membranes were immunoblotted with primary antibodies overnight at 4 °C. JP2 (NT), JP2 (CT), Calpain-1 and Calpain-2 antibodies were diluted 1:500. JP2 (M), β-Actin and α-Spectrin antibodies were diluted 1:1000. CAPNS1 and RyR2 antibodies were diluted 1:2500. Primary antibodies were then probed with secondary antibodies diluted 1:15,000 overnight at 4 °C. Proteins were visualized using the LI-COR Odyssey CLx imaging station. Protein bands of interest were quantified using the ImageJ software.

### LC-MS/MS analysis

#### Sample preparation

Protein samples were subjected to SDS-PAGE on 4–12% bis-tris minigels followed by Coomassie Blue R250 staining. Detected gel slices were cut out, reduced with 1,4-dithiothreitol, alkylated with 2-iodoacetamide, and digested overnight with trypsin. Trypsinized peptides were extracted and dried using SpeedVac (Thermo Fisher Scientific).

#### Spectral counting analysis

Peptides were enriched on a self-packed reversed-phase C18 precolumn (0.15 mm inside diameter × 20 mm, Reprosil-Pur120 C18-AQ 5 μm, Dr. Maisch), followed by separation on an analytical reversed-phase C18 column (0.075 mm inside diameter × 200 mm, Reprosil-Pur 120 C18-AQ, 3 μm, Dr. Maisch) using a 30 min linear gradient of 5 to 35% acetonitrile/0.1% formic acid (v:v) at 300 nl min^−1^. The eluent was analyzed using a Q Exactive Hybrid Quadrupole/Orbitrap Mass Spectrometer (Thermo Fisher Scientific) in data-dependent acquisition mode. Each experimental cycle consisted of an MS scan (350-1600 *m/z*, resolution setting 70,000 FWHM, AGC target 1*10e6, maximum fill time of 60 ms) and up to 12 MS/MS experiments (z=2-5, 2*10e4 trigger threshold, 15 s dynamic exclusion, 2.0 FWHM isolation width, normalized collision energy setting 25%, resolution setting 17,500 FWHM, AGC target 2*10e5, maximum fill time 60 ms). Two technical replicates per sample were acquired.

Raw data were processed for band identification in MaxQuant software version 1.5.2.8 (Max Planck Institute for Biochemistry) against a custom database containing the sequence of mouse JP2 (NP_001192005.1) using default parameters, and summarized in Scaffold software v4.89 (Proteome Software). Spectral counts were normalized to the average counts in the FL band of the undigested sample lane for each peptide.

#### Parallel reaction monitoring (PRM) analysis

Peptides were enriched on a reversed- phase pillar array trapping column (µPAC Trapping Column, Pharmafluidics), followed by separation on an analytical reversed-phase pillar array column (µPAC G1 50 cm, Pharmafluidics) using a 30 min linear gradient of 5 to 35% acetonitrile/0.1% formic acid (v:v) at 300 nl min^−1^. The eluent was analyzed using a TripleTOF 5600+ Hybrid Quadrupole/Time-of-Flight (Sciex) in data-dependent acquisition mode. For protein identification, each experimental cycle consisted of an MS scan (350–1250 resolution 35.000 FWHM, 400 ms) and up to 15 MS/MS experiments (180–1500 *m/z*, resolution 17.500 FWHM, 125 cps trigger threshold, 30 s dynamic exclusion, 0.7 FWHM isolation width, default rolling collision energy settings, 125 ms accumulation). For PRM, an additional retention time-coded inclusion list of 15 previously detected human JP2 peptides was used and dynamic exclusion disabled. Two technical replicates per sample were acquired.

Raw data were processed for peptide and protein identification in ProteinPilot software version 5.0 (Sciex) against the UniProtKB human reference proteome (revision 02-2020, 75069 entries) augmented with a set of 52 known common laboratory contaminants at using ‘thorough’ settings. Peptide quantitation from PRM data was achieved using Skyline software v20.2 (MacCoss Group, University of Washington) by extracting up to 6 fragment ion traces per peptide. Suitable peptide precursors and fragments were selected using high confidence peptide-to-spectrum (PSM) matches observed in the identification step. Peak areas were normalized to the FL band in the same lane for each peptide.

### Data and statistical analysis

Statistical significance was determined by unpaired 2-tailed t-test or 1-way-ANOVA using the mathematical analysis software GraphPad Prism 8.0, OriginLab Origin 2015 and Microsoft Excel 2010. A value of *p*<0.05 was considered statistically significant.

## Supplementary Information

Supplementary Information 1.

Supplementary Information 2.

Supplementary Information 3.

Supplementary Information 4.

## Data Availability

The mass spectrometry proteomics data have been deposited to the ProteomeXchange Consortium via the PRIDE^55^ partner repository with the dataset identifiers PXD031321 and PXD031424.
