# Supplementary material for: Calpain cleavage of Junctophilin-2 generates a spectrum of calcium-dependent cleavage products and DNA-rich NT1-fragment domains in cardiomyocytes
Source: Sci Rep. 2022 Jun 20;12:10387. doi: 10.1038/s41598-022-14320-9 (PMC9209451; doi:10.1038/s41598-022-14320-9)
Supplement: Supplementary file 4 — Supplementary Information 4. [file 41598_2022_14320_MOESM4_ESM.pdf]

Supplementary Information

Amino Acid Position	Predicted Cleavage Site	FPR (%)
52	AGVY↓TWPS	1.26
57	WPSG↓NTFE	1.25
144	YGVR↓QSVP	1.34
145	GVRQ↓SVPY	0.36
155	AVVV↓RSPL	0.77
156	VVVR↓SPLR	0.40
164	TSLS↓SLRS	0.86
167	SSLR↓SEHS	0.53
201	ALTL↓LATA	1.11
202	LTLL↓ATAE	0.48
221	TLLG↓RLRR	1.40
224	GRLR↓RSES	1.50
233	TSLG↓SQRS	0.46
243	SFLK↓SELS	0.21
247	SELS↓SGAS	0.42
257	ASTG↓SLAE	0.73
352	LPLK↓SSKV	0.92
377	ARQK↓AEIA	0.87
391	AKAK↓AEAA	1.26
482	SPAG↓TPPQ	0.33
500	DGLL↓SPGS	1.56
526	AGRR↓SPAR	1.43
548	PAPS↓QEPE	1.36
562	YHSY↓AVRT	0.67
565	YAVR↓TGPP	0.83
623	ATLE↓PKPI	1.90
646	RGLS↓KAGA	0.58
649	SKAG↓AKKK	0.99

Table S1. In silico prediction of Calpain cleavages sites in JP2 with DeepCalpain. The sequence of mouse JP2 (Uniprot: Q9ET78) was analyzed by the DeepCalpain tool predicting Calpain-specific cleavage sites. The table represents the top-28 prediction results with false positive rates (FPR) below <1.91%. Arrows indicate where the cleavage events are predicted and corresponding amino acid positions are specifically listed. Red colors indicate experimentally confirmed cleavage events.

Antibody	Epitope	Detected fragments
JP2 NT (Abcam)	66-115 (mouse)	NT_2, NT_3, FL (weak), NT_1 (weak)
JP2 M (Fitzgerald)	408-457 (human)	FL, NT_1, NT_1-NT_2 (weak), NT_1-NT_3 (very weak)
JP2 CT (Santa Cruz)	431-680 (mouse)	FL, CT_1, CT_2

Table S2. Epitope regions of different JP2 antibodies. Three different JP2 antibodies were used with affinities to different epitopes in full-length JP2 according to the manufacturers. Additionally, detected JP2 fragments after Calpain digestion are listed and weaker signals indicated.

Domain / motif	Mouse	Human
FL JP2	1-696	1-696
MORN1	14-36	14-36
MORN2	38-59	38-59
MORN3	60-79	60-79
MORN4	82-104	82-104
MORN5	106-128	106-128
MORN6	129-151	129-151
Joining region	152-284	152-290
MORN7	285-307	291-313
MORN8	308-330	313-336
α-Helical domain	351-417	357-423
Divergent region	436–664	442-664
Transmembrane domain	675-695	675-695
**Nuclear localization signal**		
Bipartite NLS	345-359	351-365
Monopartite NLS	488-492	495-499
**Predicted PEST motifs**		
PEST1	170-194 (score: 3.36)	170-197 (score: 5.37)
PEST2	243-290 (score 7.52)	250-296 (score: 6.67)
PEST3	468-488 (score: 16.37)	479-495 (score: 11.60)
PEST4	565-589 (score: 26.85)	572-610 (score: 17.03)
PEST5	590-612 (score: 17.52)	

Table S3. Domains and sequence motifs in mouse and human JP2. The domains/motifs and their sequence positions are listed for mouse JP2 (Uniprot: Q9ET78) and human JP2 (Uniprot: Q9BR39) as reported before[10,11]. PEST motifs were predicted in the mouse and human JP2 sequence with the PESTfind algorithm.

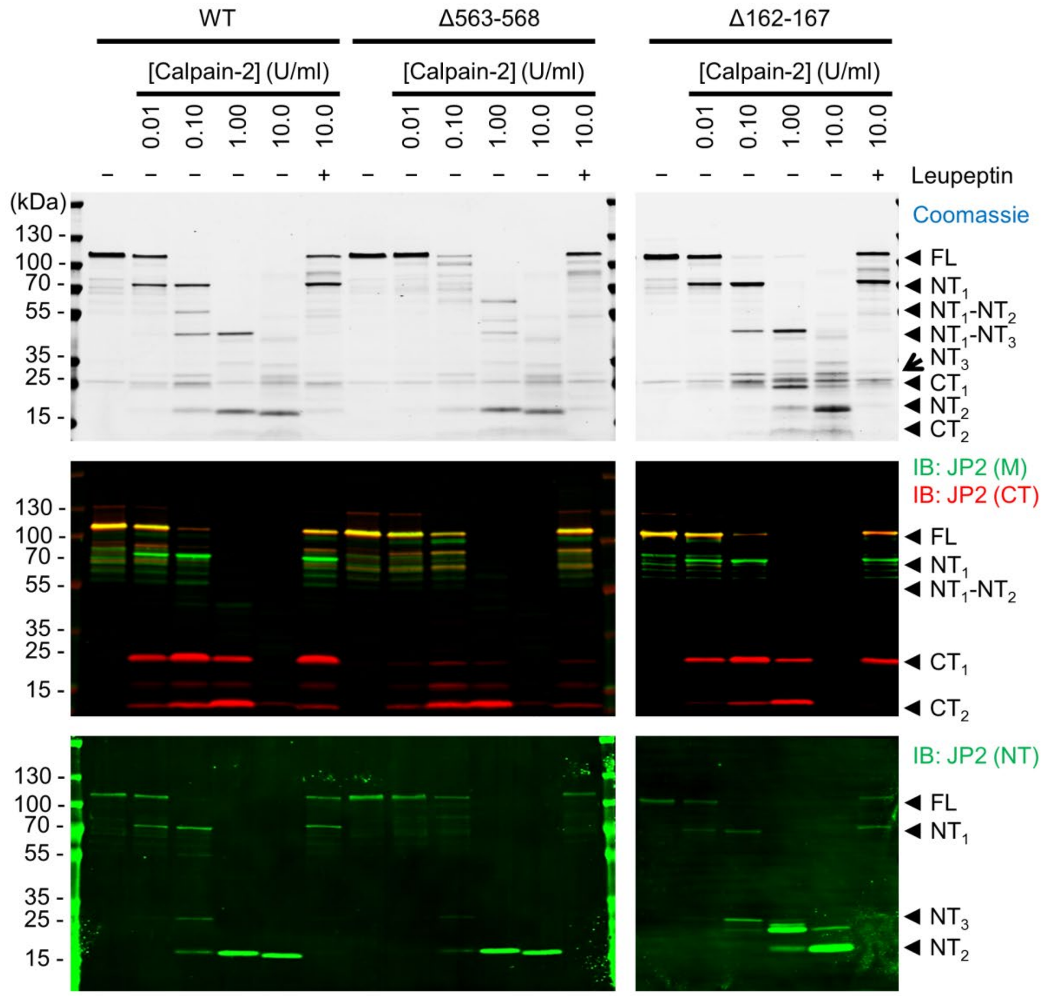

Figure S1. Calpain-2 digestion of the cleavage site-specific deletion mutants JP2$^{\Delta563\text{-}568}$ and JP2$^{\Delta162\text{-}167}$. JP2^{WT}, JP2$^{\Delta563\text{-}568}$ or JP2$^{\Delta162\text{-}167}$ were digested with increasing concentrations of Calpain-2 for 30 min at 30°C as indicated. As negative control, 10 μM Leupeptin was added to inhibit Calpain-2 at the highest concentration. Samples were subjected to SDS-PAGE. The cleavage pattern was detected by Coomassie staining (top) and immunoblotting (center & bottom) using JP2 antibodies against the N-terminal (NT), middle (M) and C-terminal region (CT). While the deletion mutation Δ563-568 prevents the 1st cleavage event, Δ162-167 prevents the 2nd cleavage event evidenced as loss of the fragments NT_1-NT_2 and NT_2. At Calpain concentrations above >0.1 U/ml, the Δ162-167 truncated protein shows additional fragments indicating further digestion of NT_3.

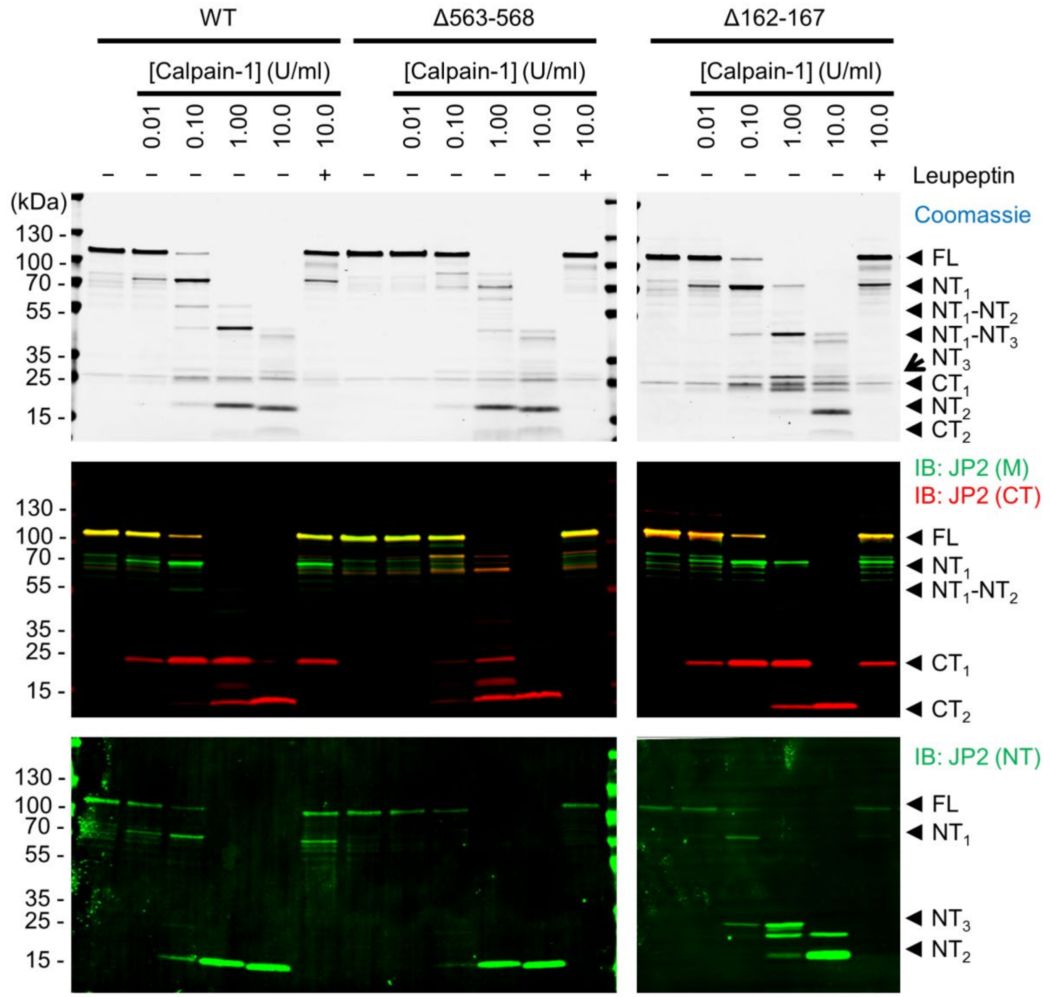

Figure S2. Calpain-1 digestion of the cleavage site-specific deletion mutants $JP2^{\Delta 563\text{-}568}$ and $JP2^{\Delta 162\text{-}167}$. $JP2^{WT}$, $JP2^{\Delta 563\text{-}568}$ or $JP2^{\Delta 162\text{-}167}$ were digested with the indicated concentrations of Calpain-1 for 30 min at 30°C. As negative control, 10 µM Leupeptin was added to inhibit Calpain-1 at the highest concentration. Samples were subjected to SDS-PAGE. The cleavage pattern was detected by Coomassie staining (top) and immunoblotting (center & bottom) using JP2 antibodies against the N-terminal (NT), middle (M) and C-terminal region (CT) as indicated. While the deletion mutation Δ563-568 prevents the 1st cleavage event, Δ162-167 prevents the 2nd cleavage event resulting in the loss of the fragments NT_1-NT_2 and NT_2. At Calpain concentrations above >0.1 U/ml, the Δ162-167 truncated protein shows additional fragments indicating further digestion of NT_3.

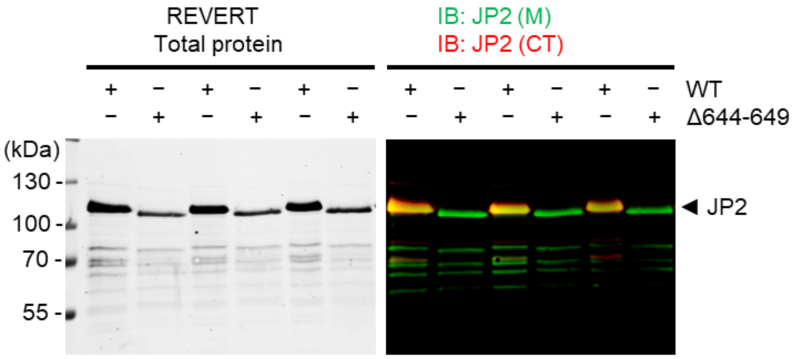

Figure S3. The JP2 deletion mutation Δ644-649 diminishes epitope detection by the C-terminal JP2 antibody. Purified $JP2^{WT}$ and the deletion mutant $JP2^{\Delta 644\text{-}649}$ were subjected to Western blot analysis. The JP2 antibody against the C-terminal region (CT) detects WT JP2 but no longer $JP2^{\Delta 644\text{-}649}$. In contrast, both the JP2 antibody against the middle region (M) and the REVERT total protein stain confirm the presence of both the $JP2^{WT}$ and $JP2^{\Delta 644\text{-}649}$ proteins. Thus, the JP2 amino acids 644-649 represent an essential region of C-terminal JP2 antibody epitope.

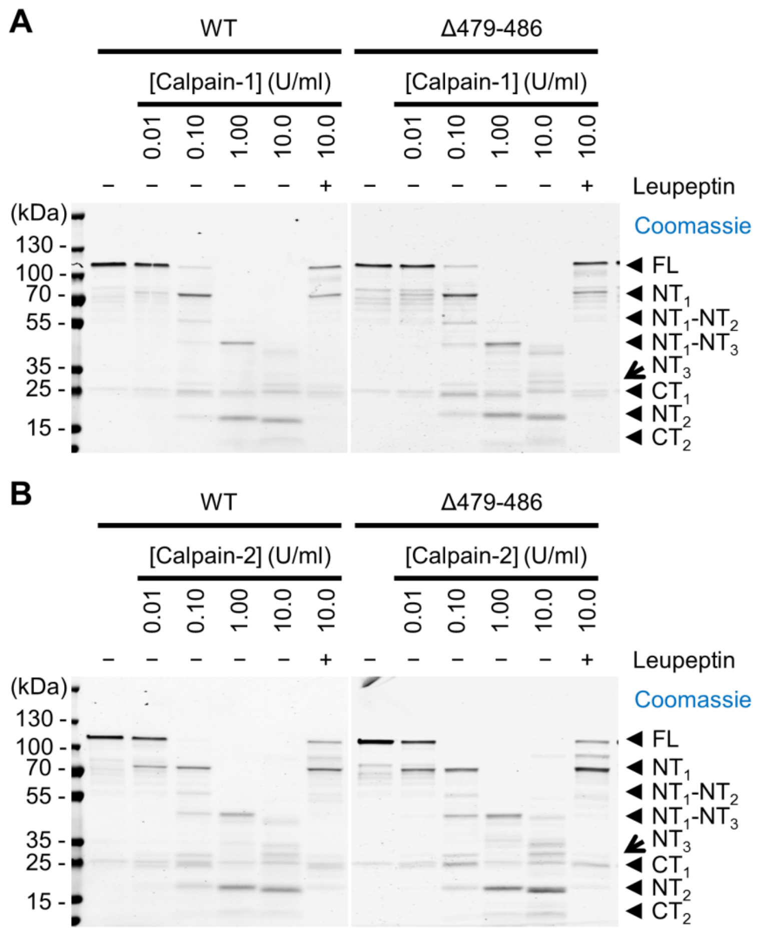

Figure S4. The JP2 deletion mutation Δ479-486 does not prevent cleavage by Calpain-1 or Calpain-2. $JP2^{WT}$ or $JP2^{\Delta 479\text{-}486}$ were digested with the indicated concentrations of Calpain-1 (**A**) or Calpain-2 (**B**) for 30 min at 30°C. As negative control, the inhibitor Leupeptin (10 µM) was added. Samples were subjected to SDS-PAGE followed by Coomassie staining. In comparison to WT, the deletion mutation Δ479-486 does not significantly alter the cleavage pattern following Calpain-1 or Calpain-2 digestion, indicating that G482 does not function as Calpain cleavage site.

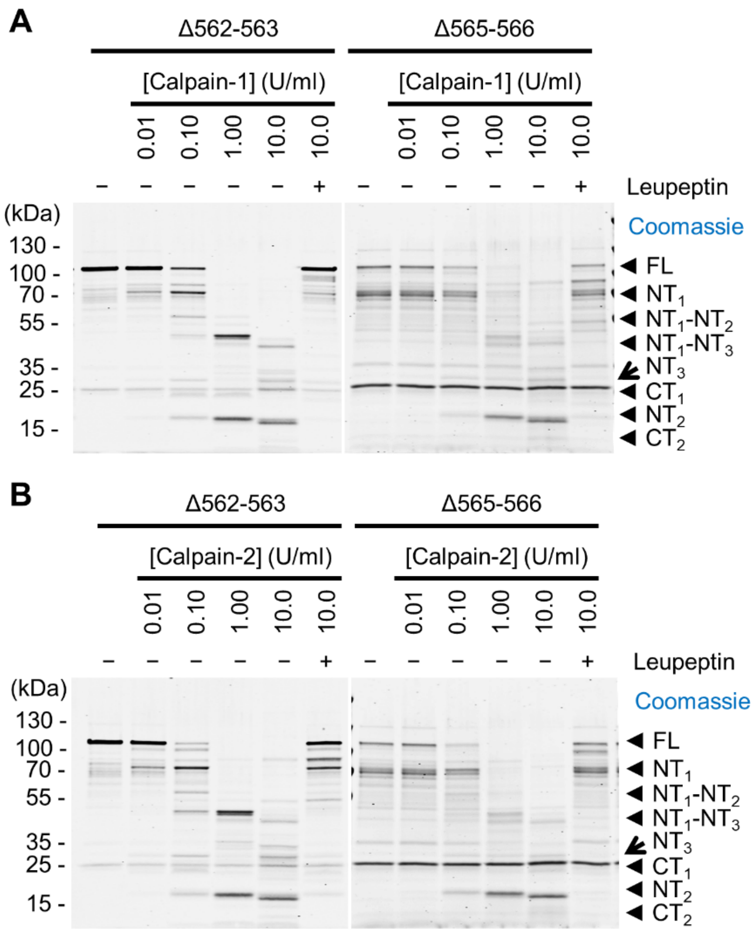

Figure S5. The initial Calpain cleavage event is stronger impaired by $JP2^{\Delta565\text{-}566}$ than by $JP2^{\Delta562\text{-}563}$. The in silico predicted (please refer to Table S1 for additional details) cleavage site deletion mutants $JP2^{\Delta562\text{-}563}$ or $JP2^{\Delta565\text{-}566}$ were digested with indicated concentrations of Calpain-1 (**A**) or Calpain-2 (**B**) for 30 min at 30°C. As negative control, the inhibitor Leupeptin (10 µM) was added. Samples were subjected to SDS-PAGE followed by Coomassie staining. The comparison shows that the initial cleavage event of JP2 by Calpain-1 or Calpain-2 is stronger impaired through the JP2 deletion mutation Δ565-566 than Δ562-563, indicating that the scissile bond is rather formed by R565-T566 than by Y562-A563.

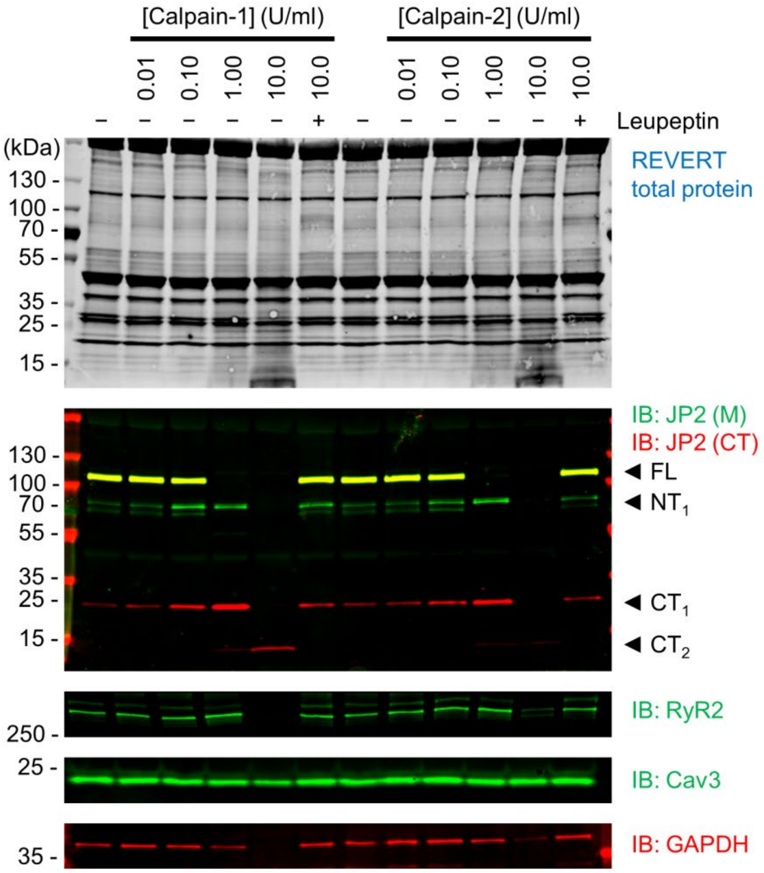

Figure S6. Proteolysis of endogenous JP2 in adult ventricular cardiomyocyte lysates by Calpain-1 and Calpain-2. Cell lysates of isolated ventricular C57BL6/N mouse cardiomyocytes were digested with the indicated concentrations of Calpain-1 or Calpain-2 for 30 min at 30°C. Samples were subjected to immunoblotting using antibodies against the middle (M) and C-terminal region (CT) of JP2, RyR2, Cav3, and GAPDH. The total protein was visualized by REVERT staining (top). The REVERT and immunostaining signal bands show that native cardiac JP2 is a substrate specifically susceptible to Calpain-mediated proteolysis compared to RyR2, Cav3 or GAPDH showing no major changes.

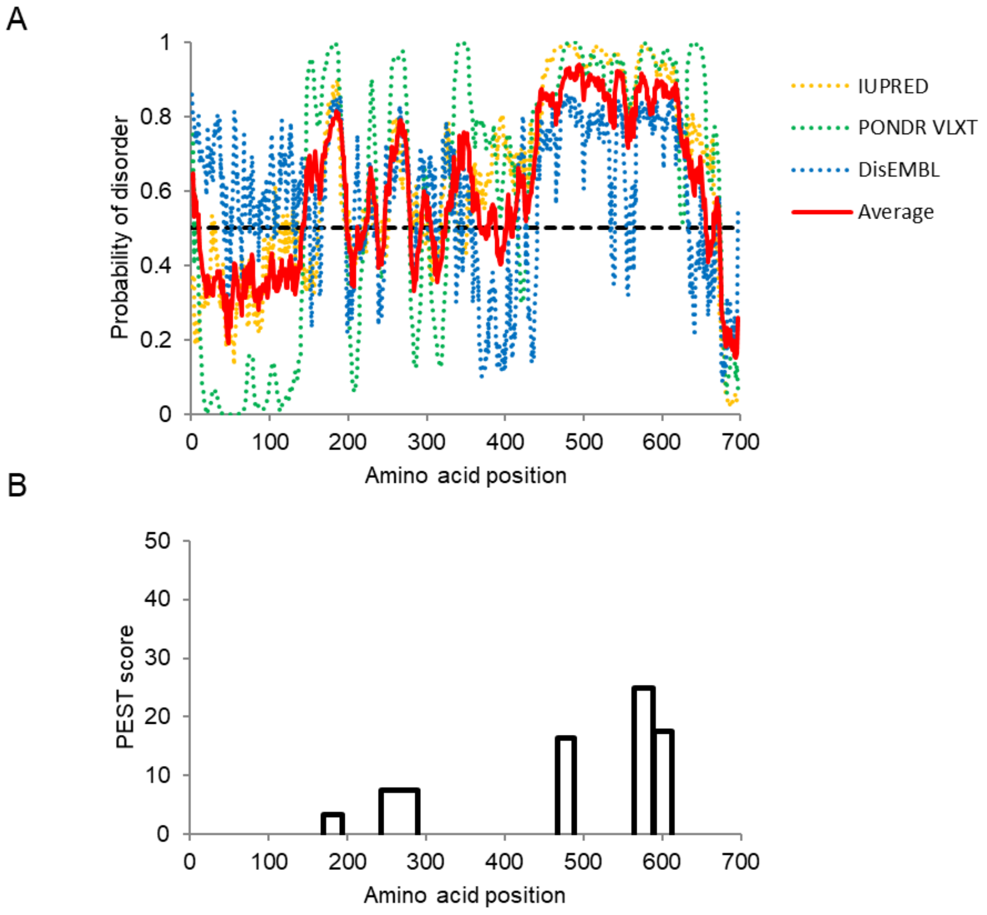

Figure S7. In silico prediction of intrinsically disordered regions and PEST motifs in JP2. (**A**) The sequence of mouse JP2 (Uniprot: Q9ET78) was analyzed using IUPRED (orange), PONDR VLXT (green), and DisEMBL (blue) algorithms. The line representing the average (red) clearly predicts the C-terminal JP2 region between amino acid position 450 and 650 as extensively intrinsically disordered region and consistently between all three algorithms. (**B**) Five potential PEST motifs were predicted in the JP2 sequence when analyzed with the PESTfind tool. The Calpain cleavage site at R565 overlaps with the highest scored PEST motif in the C-terminal JP2 region.

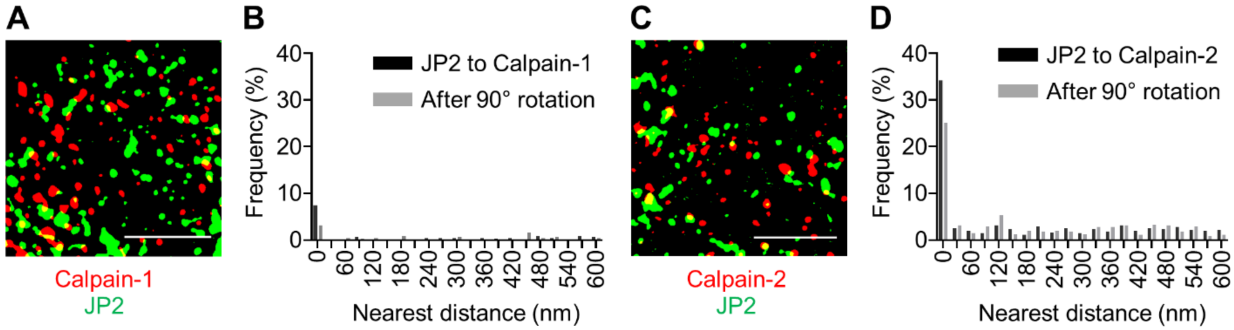

Figure S8. JP2 and Calpain-2 clusters are frequently located within nanometric proximity in hiPSC-CMs, whereas JP2 and Calpain clusters are not frequently proximity associated. (**A**, **C**) Segmented image confocal immunofluorescence data of hiPSC-CMs stained by JP2 M (green) and Calpain-1 (red) antibodies (**A**) or JP2 CT (green) and Calpain-2 (red) antibodies (**C**). Scale bar: 5 µm. (**B**, **D**) Bar graphs (black) showing the frequency distribution (%) for the indicated nearest distance bins (0 to 600 nm) for JP2 M and Calpain-1 or JP2 CT and Calpain-2, respectively. To exclude random cluster signal proximities from artificial high signal density labeling, grey bars in (**B**, **D**) compare the segmented images before and after 90° image rotation, respectively. While the JP2 and Calpain-1 cluster signals were rarely proximal to each other (d = 0 nm), the JP2 and Calpain-2 signals remained frequently in proximity as expected. Finally, we confirmed that JP2/Calpain-2 cluster proximity (d = 0 nm ≈35%) in (**D**) was significantly different after the image rotation (paired t-test, $p = 0.0002$, $n = 5$ images/hiPSC-CMs).

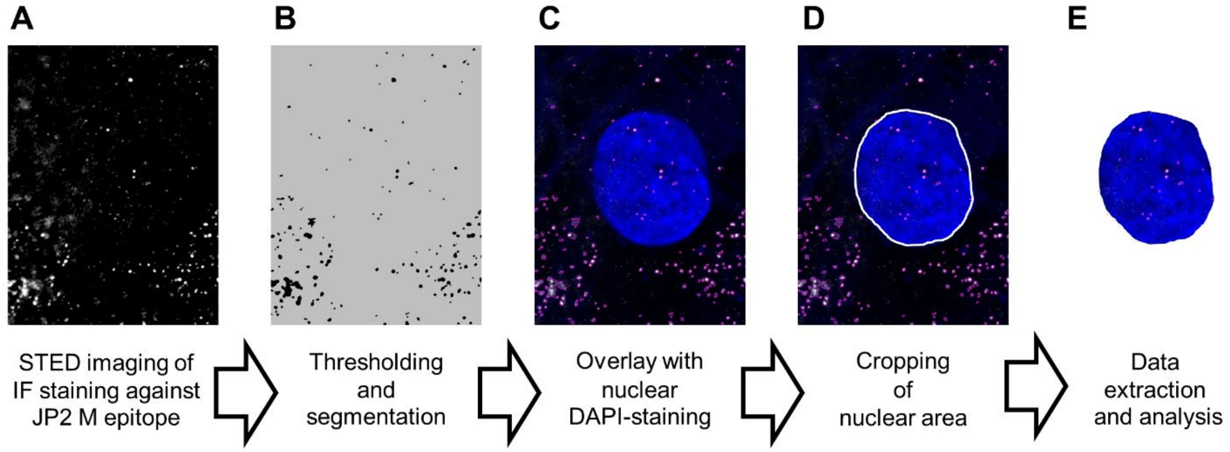

Figure S9. Workflow of intranuclear JP2 signal analysis. (**A**) STED image of hiPSC-CMs stained by immunofluorescence using the JP2 M antibody. The initial image preprocessing includes color correction, adjustment of brightness, and conversion into an 8-bit binary image. (**B**) Segmented image after Gaussian blur, background subtraction, and thresholding using the Triangle algorithm. (**C**) Overlay of segmented image with DAPI-staining (blue) and original JP2 staining (white). (**D**) Defining of the nuclear border (white line). (**E**) Determination of nuclear area and number of intranuclear JP2 signal spots.

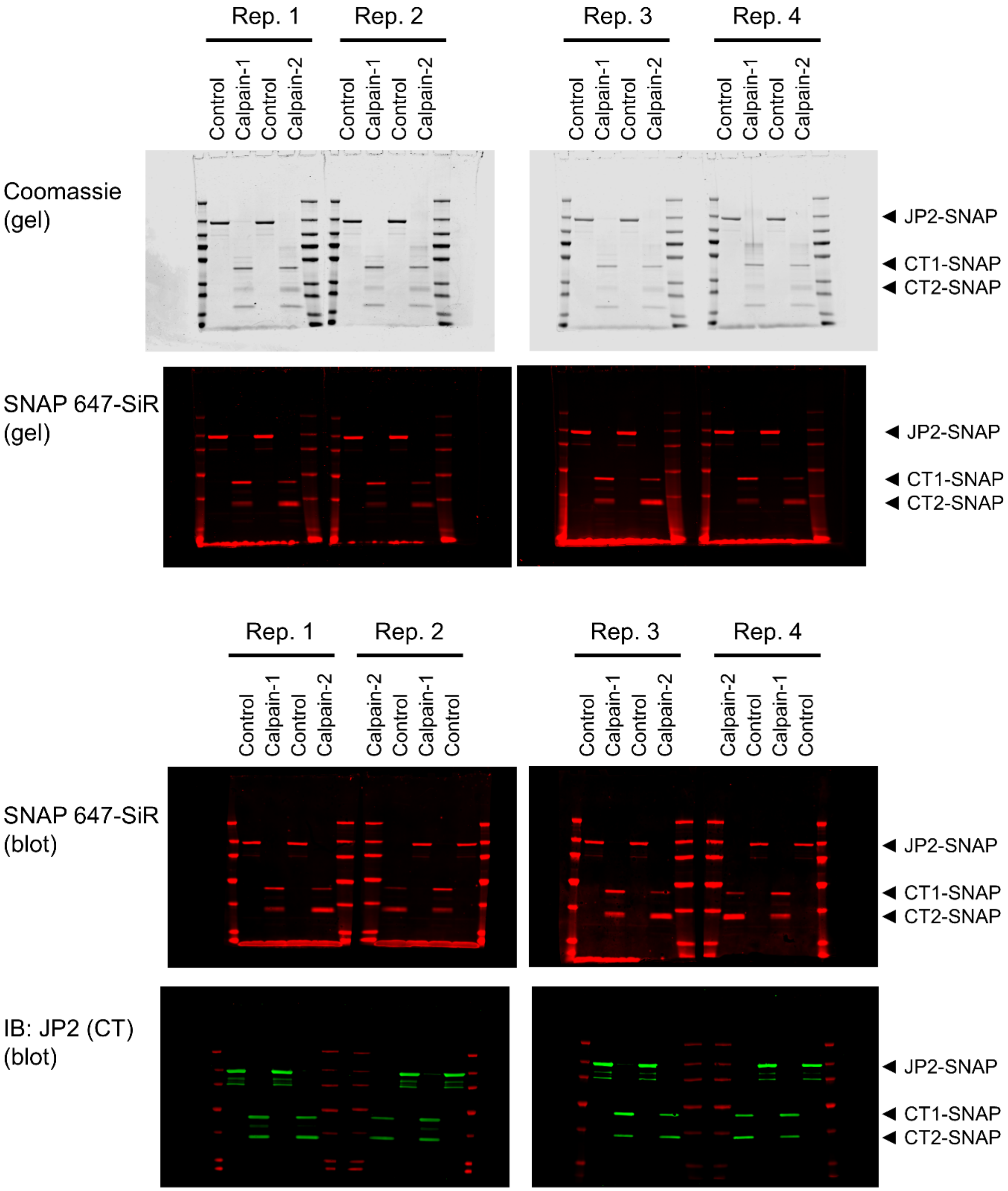

Figure S10. Original blot and gel images (1/3). Complementary original images to Fig. 3 in quadruplicate. Protein bands of interest for Fig. 3 are indicated.

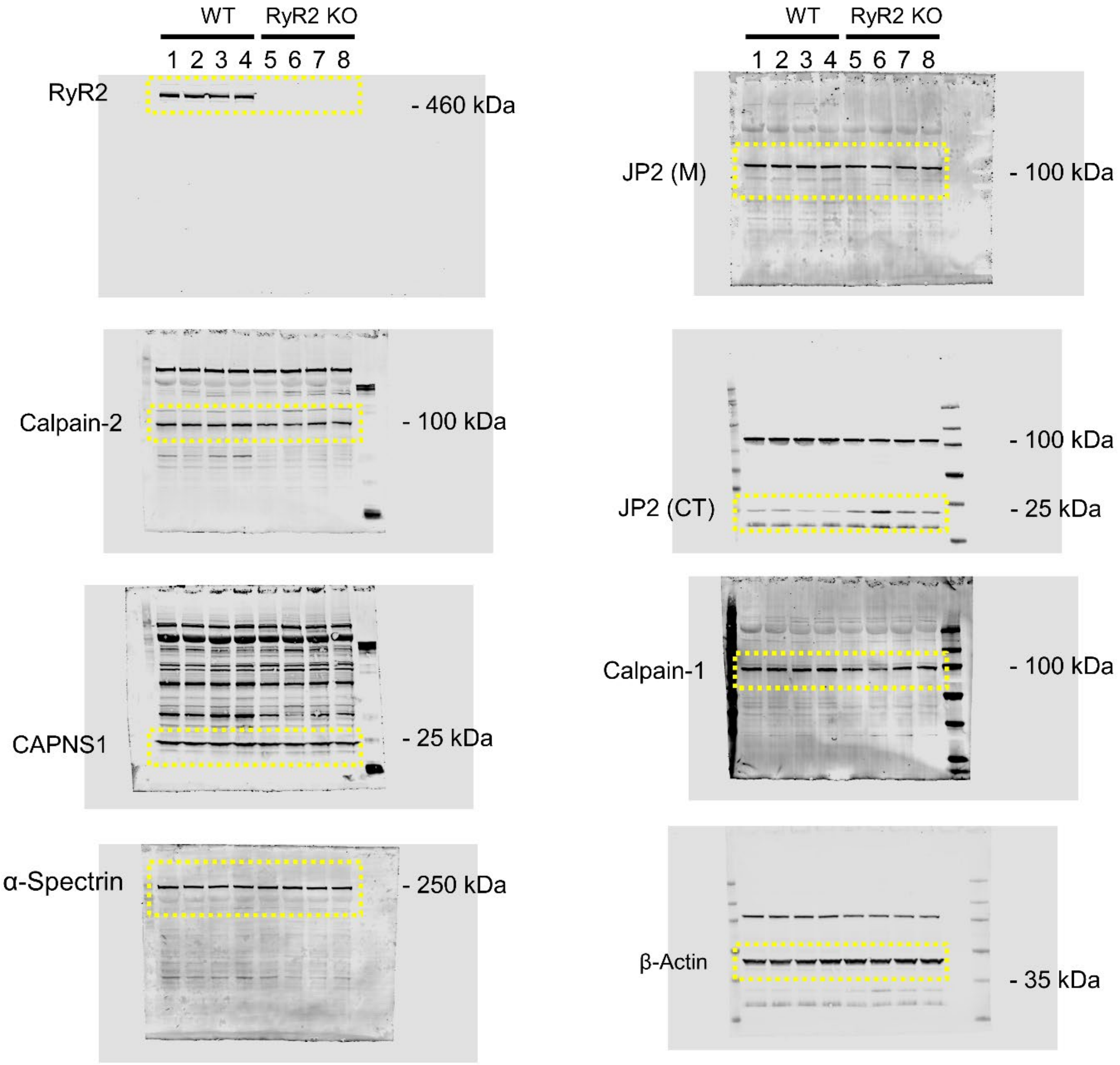

Figure S10. Original blot and gel images (2/3). Fig. 6A is based on lanes 1-8 from the original file. Protein bands of interest for Fig. 6A are indicated.

Full blots for Fig. S3

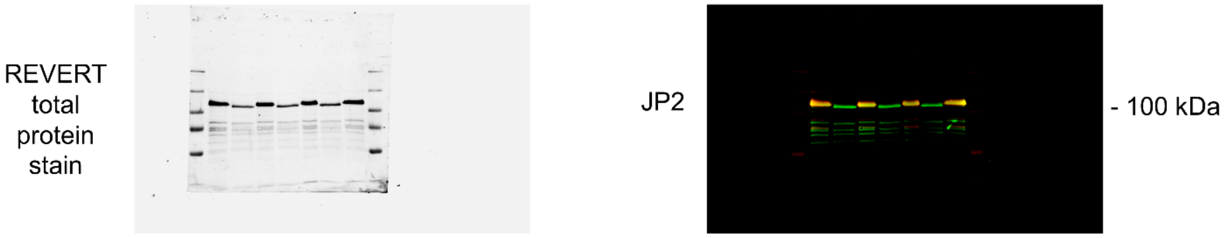

Full blots for Fig. S6

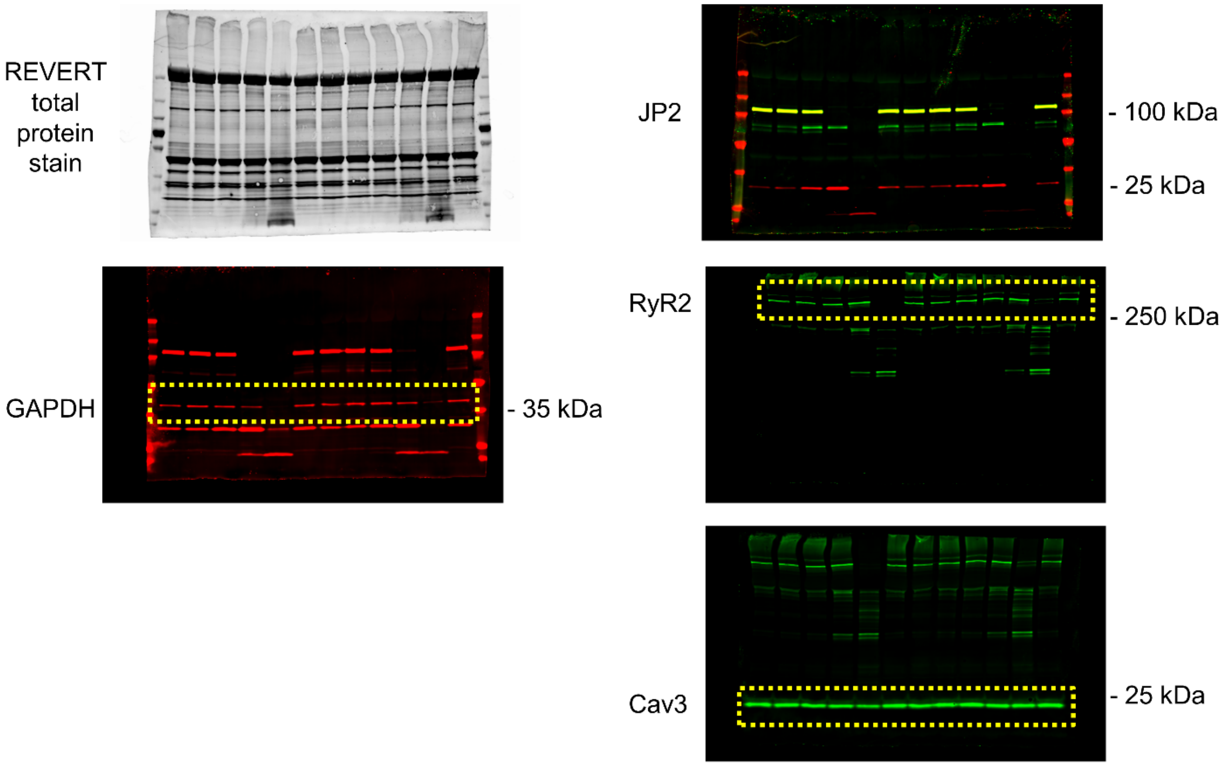

Figure S10. Original blot and gel images (3/3). Full blots for Fig. S3 and S6. Protein bands of interest for Fig. S3 and S6 are indicated.
